# Lanostane- and cycloartane-type triterpenoids from *Abies balsamea* oleoresin

**DOI:** 10.3762/bjoc.9.150

**Published:** 2013-07-04

**Authors:** Serge Lavoie, Charles Gauthier, Jean Legault, Sylvain Mercier, Vakhtang Mshvildadze, André Pichette

**Affiliations:** 1 Université du Québec à Chicoutimi, Chaire de Recherche sur les Agents Anticancéreux d'Origine Végétale, Laboratoire d'Analyse et de Séparation des Essences Végétales (LASEVE), Département des Sciences Fondamentales, 555 boul. de l'Université, Chicoutimi (Québec) G7H 2B1, Canada; 2 Université de Poitiers, Institut de Chimie IC2MP, UMR-CNRS 7285, 4 rue Michel Brunet, 86022 Poitiers, France

**Keywords:** *Abies balsamea*, cycloartane, lanostane, oleoresin, triterpenoids

## Abstract

Phytochemical analysis of *A. balsamea* oleoresin led to the isolation of three new 3,4-*seco*-lanostane triterpenoids **1**–**3**, one new cycloartane triterpenoid **4** along with fourteen known terpenoids. Structure determinations were based on extensive 1D/2D NMR, IR and MS spectroscopic analyses, and comparison with literature data. The isolated compounds were evaluated in vitro for their cytotoxicity against human cell lines (A549, DLD-1, WS1) and their antibacterial activity against *E. coli* and *S. aureus*. Abiesonic acid (**6**) exhibited weak cytotoxic activity against A549 (IC_50_ = 22 µM) while compounds **1** and **4** were weakly active against *S. aureus* (MIC = 25 µM).

## Introduction

The genus *Abies* (Pinaceae) comprises 46 species of evergreen conifers [1]. Most of them are found in temperate and boreal regions of the northern hemisphere. The first phytochemical investigation of *Abies* species was undertaken 75 years ago by Takahashi [2]. Since then, more than 277 secondary metabolites have been isolated, and mainly identified as terpenoids, flavonoids and lignans [3]. Balsam fir *Abies balsamea* (L.) Mill., a popular Christmas tree in Canada, has been used traditionally by North American aboriginal people as an antiseptic, tuberculosis remedy, and venereal aid [4]. In recent years, we have become interested in studying the bioactive constituents of *A. balsamea*. Our work allowed the identification of antibacterial sesquiterpenoids, active against *S. aureus*, from balsam fir essential oil [5]. We also isolated two cytotoxic tetraterpenoids from the cortical oleoresin of the tree bark, featuring an unprecedented C_40_ scaffold [6]. Herein, we describe the further phytochemical study of *A. balsamea* oleoresin, which led to the isolation and structure elucidation of three 3,4-*seco*-lanostane-type triterpenoids **1**–**3**, one cycloartane-type triterpenoid **4** and fourteen known terpenoids. The antibacterial (*E. coli* and *S. aureus*) and cytotoxic (A549, DLD-1 and WS1) activities of the isolated compounds are also reported.

## Results and Discussion

**Figure 1:**
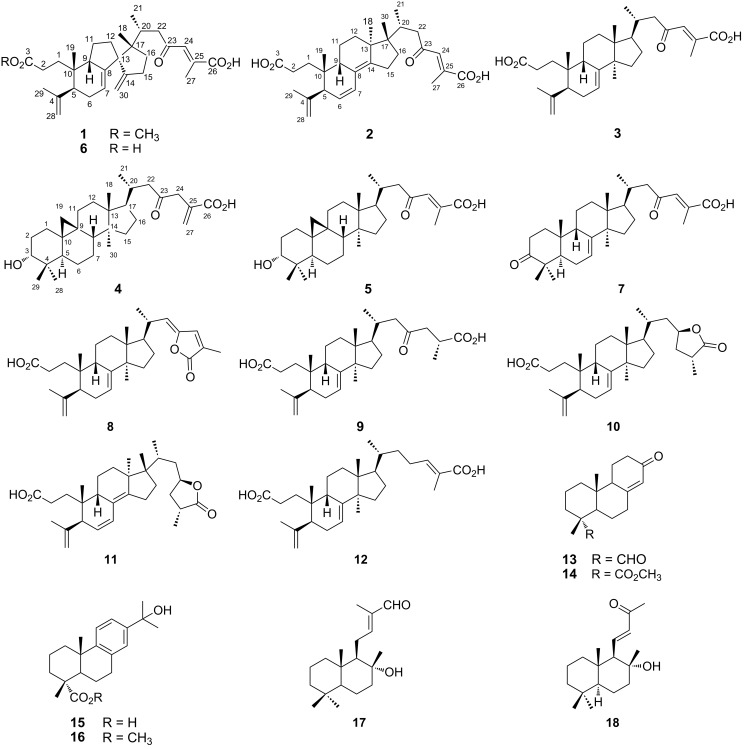
Structures of isolated compounds **1**–**18**.

The oleoresin of *A. balsamea* (1^st^ lot) was fractionated by silica gel column chromatography with hexanes/EtOAc (100:0 → 93:7) and MeOH as eluent. Both hexanes/EtOAc 93:7 and MeOH fractions were combined and concentrated under reduced pressure. Purification of this extract using a combination of silica gel or polyamide column chromatography and reversed phase C_18_ HPLC resulted in the isolation of three new (**1**–**3**) and six known terpenoids (Figure 1). In another experiment, oleoresin (2^nd^ lot) was triturated with hexanes. The precipitate was subjected to successive silica gel column chromatography followed by reversed phase C_18_ HPLC to give one new (**4**) as well as three known terpenoids. Similarly, purification of the filtrate afforded five known terpenoids. Based on their spectroscopic data (IR, MS and NMR) and comparison with literature values, the structures of the known compounds were elucidated as awashishinic acid (**5**) [7], abiesonic acid (**6**) [6], firmanoic acid (**7**) [8], (22*Z*)-3,4-*seco*-9β*H*-lanosta-4(28),7,22,24-tetraen-23,26-olid-3-oic acid (**8**) [9], (25*R*)-3,4-*seco*-9β*H*-lanosta-4(28),7-diene-3,26-dioic acid (**9**) [10], abiesolidic acid (**10**) [10–11], (23*R*,25*R*)-3,4-*seco*-17,14-*friedo*-9β*H*-lanosta-4(28),6,8(14)-trien-26,23-olid-3-oic acid (**11**) [10], (24*E*)-3,4-*seco*-9β*H*-lanosta-4(28),7,24-triene-3,26-dioic acid (**12**) [12], abiesanordine C (**13**) [13], methyl 13-oxo-podocarp-8(14)-en-15-oate (**14**) [14], 15-hydroxydehydroabietic acid (**15**) [15], methyl 15-hydroxydehydroabietate (**16**) [16], (12*E*)-8-hydroxy-15-nor-12-labden-14-al (**17**) [17] and 8-hydroxy-14,15-dinor-11-labden-13-one (**18**) [13,18] (Figure 1). ^1^H and ^13^C NMR spectroscopic data of known compounds (**5**–**18**) are given in Supporting Information File 1.

Compound **1** was isolated as a white amorphous powder. Its molecular formula was established as C_31_H_44_O_5_ from the [M + H]^+^ peak at *m*/*z* 497.3261 (calcd 497.3262) in the positive HRESIMS, indicating ten degrees of unsaturation. The IR spectrum displayed strong absorption bands at 1692 and 1736 cm^−1^ indicative of carboxylic acid functionalities. The ^13^C NMR and DEPT spectroscopic data (Table 1) exhibited 31 carbons including one carbonyl carbon at δ_C_ 202.4, and two carboxylic carbons at δ_C_ 172.4 and 174.8. The ^1^H NMR data (Table 2) exhibited six olefinic signals at δ_H_ 4.73 (s), 4.77 (s), 4.78 (s), 4.86 (s), 5.48 (dd, *J =* 6.2, 3.1 Hz) and 7.11 (br s), one methoxy methyl at δ_H_ 3.67 (s), four tertiary methyl at δ_H_ 0.90 (s), 0.92 (s), 1.75 (s) and 2.18 (s) and one secondary methyl at δ_H_ 0.85 (d, *J* = 6.4 Hz). Detail analysis of the above NMR information, together with ^1^H–^1^H COSY, HSQC and HMBC analyses indicated that **1** shares the same structure with abiesonic acid (**6**), previously isolated from *A. balsamea* [6], but with an additional methoxy group. An HMBC cross-peak between this methyl signal and the carbon at δ_C_ 174.8 (C-3) allowed the assignment of compound **1** as (−)-*rel-*abiesonic acid 3-methyl ester.

**Table 1:** ^13^C NMR spectroscopic data (100 MHz, CDCl_3_) of compounds **1**–**4**.

Position	**1**	**2**	**3**	**4**

1	30.5	28.3	28.8	27.5
2	29.2	29.8	29.2	28.5
3	174.8	181.6	180.8	77.1
4	149.2	145.6	149.7	39.5
5	44.0	50.6	45.3	41.1
6	30.9	127.0	29.7	21.1
7	122.4	125.2	118.0	25.6
8	143.4	125.4	146.3	48.0
9	49.5	39.4	38.6	19.7
10	36.9	37.0	36.3	26.5
11	22.5	19.6	18.5	26.2
12	31.2	32.0	33.8	32.8
13	63.5	47.4	43.8	45.4
14	160.9	146.2	51.7	49.0
15	27.8	23.9	34.0	35.4
16	36.1	36.3	28.5	28.3
17	50.2	49.1	53.1	52.2
18	17.7	21.9	21.7	18.1
19	24.7	21.8	24.1	29.8
20	33.8	35.1	33.3	32.9
21	16.4	15.9	19.5	19.3
22	48.3	48.9	51.9	50.0
23	202.4	202.5	202.4	207.6
24	134.9	133.0	134.4	46.1
25	138.7	140.4	139.3	133.9
26	172.4	173.4	172.8	170.8
27	14.0	14.0	13.9	130.5
28	111.9	115.6	112.0	25.8
29	26.1	24.8	26.0	21.2
30	106.9	15.8	27.5	19.3
OMe	51.7	–	–	–

**Table 2:** ^1^H NMR spectroscopic data (400 MHz, CDCl_3_) of compounds **1**–**4**.

Position	δ_H_ (*J* in Hz)			

	**1**	**2**	**3**	**4**

1	1.74, m, 1.62, m	1.60, m	1.73, m, 1.60, m	1.85, m, 1.01, m
2	2.30, m	2.31, m	2.32, m	1.93, m, 1.64, m
3	–	–	–	3.48, t (2.4)
5	2.08, m	2.63, d (5.4)	2.08, m	1.82, m
6	2.40, m, 2.13, m	5.39, dd (9.9, 5.5)	2.27, m, 1.99, m	1.48, m, 0.77, m
7	5.48, dd (6.2, 3.1)	6.22, d (10.0)	5.33, br s	1.30, m, 1.11, m
8	–	–	–	1.54, m
9	2.06, m	2.43, m	2.59, m	–
11	1.59, m, 1.40, m	1.62, m	1.60, m	2.00, m, 1.13, m
12	1.77, m, 1.32, m	1.65, m	1.83, m, 1.67, m	1.62, m
15	2.48, m, 2.37, m	2.41, m, 2.32, m	1.52, m	1.31, m
16	1.55, m	1.73, m, 1.54, m	1.92, m, 1.26, m	1.87, m, 1.27, m
17	–	–	1.54, m	1.61, m
18	0.90, s	1.16, s	0.80, s	1.00, s
19	0.92, s	0.87, s	0.86, s	0.52, d (3.9), 0.35, d (3.9)
20	2.39, m	2.24, m	2.03, m	2.02, m
21	0.85, d (6.4)	0.80, d (6.5)	0.91, d (6.2)	0.88, d (6.8)
22	2.49, m, 2.25, m	2.85, m 2.16, br d (12.3)	2.64, m 2.32, m	2.56, dd (16.0, 2.1), 2.24, dd (16.1, 10.2)
24	7.11, br s	7.23, br s	7.15, s	3.42, d (17.0) 3.36, d (17.1)
27	2.18, s	2.22, d (1.0)	2.21, s	6.45, br s 5.73, br s
28	4.86, s, 4.78, s	4.98, br s, 4.76, d (2.4)	4.88, s, 4.82, s	0.95, s
29	1.75, s	1.79, s	1.80, s	0.88, s
30	4.77, s, 4.73, s	0.69, s	1.04, s	0.90, s
OMe	3.67, s	–	–	–

**Figure 2:**
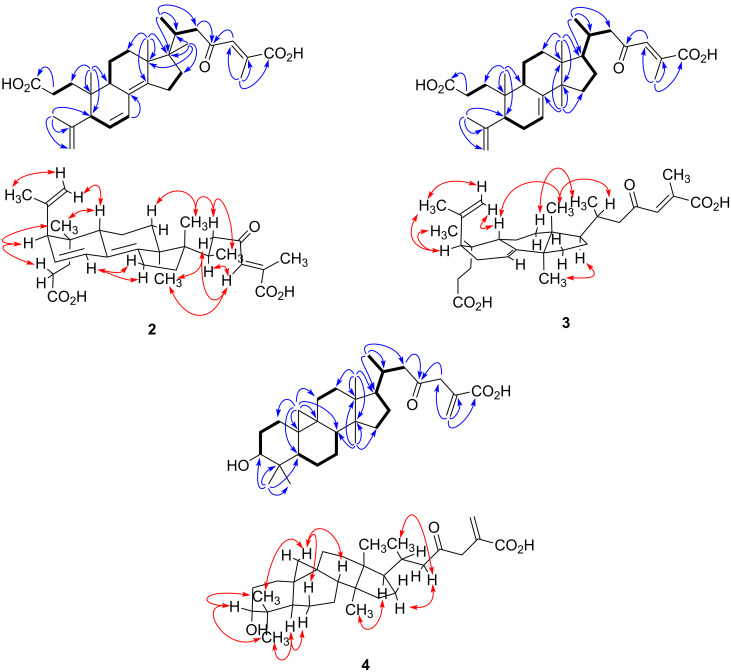
Selected COSY (▬), HMBC (blue arrows) and NOESY (red arrows) correlations for compounds **2**–**4**.

Compound **2**, obtained as a white amorphous powder, possessed a molecular formula of C_30_H_42_O_4_ with ten degrees of unsaturation based on the [M + H]^+^ peak at *m*/*z* 483.3087 (calcd 483.3105) in the positive HRESIMS. The IR absorption bands showed the presence of carboxylic acid (1702 cm^−1^) and olefin (1635 cm^−1^) functionalities. The ^13^C NMR spectroscopic data of **2** (Table 1) displayed 30 carbon signals, which by the assistance of a DEPT experiment, were identified as six methyl, seven *sp*^3^ methylene and three *sp*^3^ methine groups, three *sp*^3^ quaternary carbon atoms, one *sp*^2^ methylene and three *sp*^2^ methine groups, and seven *sp*^2^ quaternary carbon atoms. A ^1^H–^1^H COSY experiment provided correlations from H_2_-1 to H_2_-2, H-6 to H-5 and H-7, H_2_-11 to H-9 and H_2_-12, H_2_-15 to H_2_-16 and H-20 to H_3_-21 and H_2_-22 (Figure 2). Analysis of HMBC spectra indicated correlations from H_3_-19 to C-1, C-5, C-9 and C-10; from H_3_-29 to C-4, C-5 and C-28; from H-7 to C-8; from H_3_-18 to C-12, C-13, C-14 and C-17; from H_3_-30 to C-13, C-16, C-17 and C-20; from H_3_-21 to C-17, C-20 and C-22; from H_2_-22 and H-24 to C-23; and from H_3_-27 to C-24, C-25 and C-26. The relative configuration of **2** was determined by analysis of a NOESY experiment, which provided correlations (Figure 2) of H-5 to H_2_-2; H-28Z to H-9; H-22a (δ_H_ 2.85) to H_3_-18 and H_3_-21; H_3_-18 to H-22b (δ_H_ 2.16) and H-24; H-24 to H-20 and H-22b. These correlations indicated the α-orientation of H-5 and H_3_-30 and the β-orientation of H-9, H_3_-18 and H_3_-19. All these facts suggested that compound **2** was strongly similar to *cis*-sibiric acid [19]. Since the chemical shift of H-24 in *cis*-sibiric acid (δ_H_ 6.15) was upfield of the signal for **1** (δ_H_ 7.11), **2** (δ_H_ 7.23), **6** (δ_H_ 7.13) and **7** (δ_H_ 7.07), this suggested that the *trans*-stereoisomer was isolated instead of the *cis*-one (See Table 2 and Supporting Information File 1). This was further confirmed by NOESY correlation of H-24 to H-20 and H_3_-30, but not to H_3_-27. Consequently, the structure of **2** was determined as (−)-*rel-*(24*E*)-23-oxo-3,4-*seco*-9β*H*-lanosta-4(28),6,8(14),24-tetraen-3,26-dioic acid.

Compound **3**, a white amorphous powder, possessed a molecular formula of C_30_H_44_O_5_ based on the [M + H]^+^ peak at *m*/*z* 485.3250 (calcd 485.3262) in the positive HRESIMS, suggesting nine degrees of unsaturation. The IR spectrum implied the existence of carboxylic acid (1703 cm^−1^) and olefin (1633 cm^−1^) functionalities. The ^13^C NMR spectroscopic data of **3** resembled those of (24*E*)-3,4-*seco*-9β*H*-lanosta-4(28),7,24-triene-3,26-dioic acid (**12**) [12] except for change at δ_C_ 33.3 (C-20), 19.5 (C-21), 51.9 (C-22), 202.4 (C-23), 134.4 (C-24), 139.3 (C-25), 172.8 (C-26) and 13.9 (C-27) (See Table 1 and Supporting Information File 1). The HMBC correlations from H-24 to C-23 indicated the presence of a ketone group at C-23 (Figure 2). This conclusion was confirmed from the downfield δ_C_ of C-22 (+16.4) in comparison with **12**. The relative configuration was established with the NOESY spectrum (Figure 2). Briefly, the configuration at C-5, C-9, C-10, C-13 and C-17 was determined by cross-peaks from H-28Z to H-9; H-5 to H_3_-19 and H_3_-29; H_3_-18 to H-9 and H-20; H_3_-30 to H-17; and H_3_-21 to H_2_-12. NOESY correlation between H-24 and H_3_-27 was not observed, suggesting that the geometry of the C-24,25 double bond was *E*. This was confirmed by δ_H_ comparison of H-24 with that of **1**, **2**, **6** and **7** (See Table 2 and Supporting Information File 1). On the basis of these spectroscopic evidences, the structure of **3** was assigned as (−)-*rel-*(24*E*)-23-oxo-3,4-*seco*-9β*H*-lanosta-4(28),7,24-triene-3,26-dioic acid.

The HRESIMS of **4**, isolated as a white amorphous powder, showed a pseudomolecular [M + H]^+^ ion peak at *m*/*z* 471.3463, corresponding to the formula C_30_H_46_O_4_ (calcd. 471.3469), indicating eight degrees of insaturation. The IR absorption bands at 3416, 1708 and 1633 cm^−1^ suggested the presence of hydroxyl, carbonyl and olefin functionalities. The ^13^C NMR and DEPT-135 spectra of **4** showed signals for 30 carbons designated as five methyl; twelve methylene, including one alkene at δ_C_ 130.5; five methine, including one secondary alcohol at δ_C_ 77.1; and eight quaternary carbons, including those at δ_C_ 170.8 and 207.6 representing carboxylic and ketone carbonyls, respectively (Table 1). The ^1^H NMR spectrum showed two doublets at δ_H_ 0.35 (*J* = 3.9 Hz) and 0.52 (*J* = 3.9 Hz) characteristic of a cyclopropane ring (Table 2), suggesting that **4** is a member of the cycloartanes, which is an important triterpenic family in the genus *Abies* [3]. In the ^1^H–^1^H COSY spectrum, correlations between H_2_-2 to H_2_-1 and H-3; H_2_-6 to H-5 and H_2_-7; H_2_-7 to H-8; H_2_-16 to H_2_-15 and H-17; and H-20 to H_3_-21 and H_2_-22 were observed (Figure 2). HMBC correlations from H_2_-19 to C-1, C-5, C-8, C-9, C-10 and C-11 connected together three different fragments in the vicinity of the cyclopropyl group. Other correlations between H_3_-18 to C-12, C-13, C-14 and C-17; H_3_-21 to C-17, C-20 and C-22; H_2_-27 to C-24, C-25 and C-26; H_3_-28 and H_3_-29 to C-3, C-4, C-5, C-28 and C-29; H_3_-30 to C-8, C-13, C-14 and C-15; and H_2_-22 and H_2_-24 to C-23 were observed and completely assigned the carbon skeleton of the molecule (Figure 2). The relative configuration was determined with the help of a 2D NOESY experiment showing correlations from H-19β to H-6β, H-8 and H_3_-29; H-5 to H_3_-28 and H-6α; H_3_-30 to H-11α and H-17; and H-22b to H-20 and H_2_-16 (Figure 2). The α-orientation of the hydroxy group at C-3 was deduced from the small coupling constant of H-3 (*J* = 2.4 Hz), and from the NOESY correlations with both H_3_-28 and H_3_-29. Accordingly, the structure of compound **4** was defined as (+)-*rel-*3α-hydroxy-23-oxocycloart-25(27)-en-26-oic acid.

The absolute stereochemistry of the new compounds (**1**–**4**) has not been determined experimentally. However, the previously described compounds **7**, **9**, **10** and **11** have been shown to possess the usual configuration for triterpenes [8,10–11]. The structures of many other triterpenes isolated from the genus *Abies* were also reported with this absolute configuration according to their X-ray crystallographic data [20–22].

The structure of compound **8** was reported by Xia et al [9]. In their paper, the configuration at Δ^22^ was determined as *E* but it was not supported by any spectroscopic data. Since ^1^H and ^13^C NMR data obtained for **8** were identical to those reported by Xia within 0.01 and 0.1 ppm respectively (see Supporting Information File 1), we supposed that both molecules were the same. However, the geometry at Δ^22^ should be assigned to *Z* because of the clear NOESY correlation between H-22 and H-24. Interestingly, lanostane with *E* geometry at Δ^22^ has never been isolated so far. Moreover, triterpenes with this kind of side chain bearing an *E* configuration for Δ^22^ have only been reported by Guo et al [23–24]. During their work on *Schisandra* spp., they isolated many nortriterpenes having both Δ^22^ configurations. A statistical analysis of the ^1^H chemical shift for H-22 and H-24 was conducted: for *E*-configured Δ^22^, δ_H_ are 5.9 ± 0.2 and 7.8 ± 0.1 while for *Z*-configured Δ^22^, δ_H_ are 5.3 ± 0.1 and 7.2 ± 0.2, respectively. Since δ_H_ measured for compound **8** was 4.98 and 6.97, it should be assigned as (22*Z*)-3,4-*seco*-9β*H*-lanosta-4(28),7,22,24-tetraen-23,26-olid-3-oic acid.

The isolates were evaluated in vitro for their cytotoxic activities against two human cancer cell lines, namely lung carcinoma (A549) and colon adenocarcinoma (DLD-1), as well as against one healthy cell line (WS1) using the resazurin reduction test [25]. Etoposide was used as a positive control (IC_50_ ≤ 1.0 µM). None of the compounds were found to be active (IC_50_ > 25 µM) with the exception of abiesonic acid (**6**), which showed a weak cytotoxic activity against A549 (IC_50_ = 22 µM). The antibacterial activity of isolated compounds was also evaluated in vitro against *E. coli* and *S. aureus* using the microdilution assay [26] with gentamycin as a positive control (MIC < 0.1 µg/mL). No activity was observed for all the tested compounds (MIC ≥ 50 µM) except for triterpenoids **1** and **4**, which were weakly active against *S. aureus* (MIC = 25 µM).

## Supporting Information

File 1 Experimental procedures, product characterization and ^1^H and ^13^C spectra for compounds **1**–**18**.
