# Supplementary material for: Lanostane- and cycloartane-type triterpenoids from Abies balsamea oleoresin
Source: Beilstein J Org Chem. 2013 Jul 4;9:1333–9. doi: 10.3762/bjoc.9.150 (PMC3740800; doi:10.3762/bjoc.9.150)

Supporting information

for

Lanostane- and cycloartane-type triterpenoids from *Abies balsamea* oleoresin

Serge Lavoie[1,§], Charles Gauthier[1,2,§], Jean Legault[1], Sylvain Mercier[1], Vakhtang Mshvildadze[1] and André Pichette[1,*]

Address: [1]Université du Québec à Chicoutimi, Chaire de Recherche sur les Agents Anticancéreux d'Origine Végétale, Laboratoire d'Analyse et de Séparation des Essences Végétales (LASEVE), Département des Sciences Fondamentales, 555 boul. de l'Université, Chicoutimi (Québec) G7H 2B1, Canada and [2]Université de Poitiers, Institut de Chimie IC2MP, UMR-CNRS 7285, 4 rue Michel Brunet, 86022 Poitiers, France

[§]These authors contributed equally to this work.

Email: André Pichette - andre_pichette@uqac.ca

* Corresponding Author

Experimental procedures, product characterization and ^{1}H and ^{13}C spectra for compounds 1–18

Experimental

General methods

Optical rotations $[\alpha]^{20}_D$ were measured on an Autopol IV polarimeter. FTIR spectra were recorded on a Perkin-Elmer SpectrumOne. For the purpose, the sample was deposited on a NaCl window using $CHCl_3$ solution and dried before analysis. The 1D (^1H, ^{13}C and DEPT135) and 2D NMR spectra (^1H–^1H COSY, HSQC, HMBC and NOESY) were performed using an Avance 400 Bruker spectrometer (400.13 MHz for ^1H, 100.61 MHz for ^{13}C) equipped with a 5 mm QNP-probe. All spectra were acquired in CD_3OD, $CDCl_3$, $CD_3OD/CDCl_3$ 1:1 or pyridine-d_5 and chemical shifts are reported in ppm (δ) relative to TMS. High resolution electrospray ionization mass spectra (HRESIMS) were obtained in positive mode on an Agilent 6210 TOF LCMS. HPLC-APCI MS (negative mode) were conducted on an Agilent 1100 series system consisting of a degasser, a quaternary pump, an automatic injector, a temperature-controlled column compartment, a diode array detector and a mass selective detector Agilent G1946 VL model equipped with an APCI source. Analytical separations were performed on a 6.0 × 250 mm Inertsil prep-ODS C_{18} reversed-phase column (10 µm of particle size) using a H_2O/CH_3CN system with the pH of water adjusted to ~4 with formic acid (HPLC grade) to improve peak sharpness. Chromatographic conditions were the following: isocratic elution with H_2O pH 4/CH_3CN (30:70 to 5:95) at a flow rate of 0.8 mL/min and column oven at 25 °C. Preparative HPLC separation (Agilent 1100) were carried out on a 20.0 × 250 mm Inertsil prep-ODS C_{18} column using a multiple-wavelength detector and an automatic fraction collector. The solvents were purchased from VWR (Canada). The TLC plates (aluminium sheets of silica gel ultra-pure 250 µm, with indicator F_{254}) and silica gel ultra-pure (40–63 µm with indicator F_{254}) were supplied from Silicycle (Canada) and low-pressure liquid chromatography columns were purchased from Büchi Labortechnik (Suisse). Polyamide CC-6 was purchased from Macherey-Nagel (Germany). Solvent systems for TLC analyses were: (a) hexanes/EtOAc (3:1), (b) $CHCl_3$/EtOAc (80:1 to 20:1), (c) $CHCl_3$/MeOH (40:1) developing with H_2SO_4 (20% in MeOH) with charring at 100 °C.

Plant material

Oleoresin (1st lot) was harvested by M. Marcel Pichette in Summer 2007 at Saguenay, Québec, Canada. A specimen of the scored tree was identified by M. Patrick Nadeau and submitted to herbium Louis-Marie at Université Laval (QFA0579436). In the second purification protocol, the oleoresin (lot number: GS0911, 2nd lot) was provided by Les Gommes de Sapin du Québec, Inc., Charlevoix (Québec), Canada.

Extraction and isolation

Extraction and isolation of compounds **1**–**3**, **6**, **8**, **10**, **11** and **15** were achieved in a similar manner as described by Lavoie et al. [1], *Abies balsamea* (L.) Mill. (Pinaceae) oleoresin (500 g, 1st lot) was directly fractionated by silica gel column using hexanes/EtOAc (100:0 → 93:7) as eluant and finally washed by MeOH. The fractions obtained with hexanes/EtOAc 93:7 and MeOH were combined and the resulting solution was evaporated under reduced pressure to obtain 75 g of a light brown gum. A part of this extract (Fr A, 60 g) was further fractionated by silica gel chromatography eluting with hexanes/EtOAc 3:1 (A.1, 14 g), hexanes/EtOAc 2:1 (A.2, 19 g), hexanes/EtOAc 1:1 and finally washed with MeOH (A.3, 21.5 g). Subfraction A.2 was fractionated by silica gel column with hexanes/EtOAc (3:1) giving five other subfractions A.2.1–A.2.5. Subfraction A.2.3 was further chromatographed on silica gel column with $CHCl_3$/MeOH (40:1) yielding three subfractions A.2.3.1–A.2.3.3. Fraction A.2.3.2 was purified on RP18 flash chromatography using gradient elution (H_2O/MeOH 20:80 → 0:100) followed by HPLC (H_2O/ACN 20:80) giving abiesonic acid 3-methyl ester (**1**, 12.1 mg). Fraction A.2.3.3 was purified on RP18 flash chromatography using gradient elution (H_2O/MeOH 20:80 → 0:100). Further HPLC separation (H_2O-ACN 20:80) afforded (22*Z*)-3,4-*seco*-9β*H*-lanosta-4(28),7,22,24-tetraen-23,26-olid-3-oic acid (**8**, 15.2 mg), abiesolidic acid (**10**, 18.5 mg) and (23*R*,25*R*)-3,4-*seco*-17,14-*friedo*-9β*H*-lanosta-4(28),6,8(14)-trien-26,23-olid-3-oic acid (**11**, 14.3 mg). Purification of A.2.5 on Polyamide column (H_2O/MeOH 20:80) and further preparative HPLC separation (H_2O/MeOH 20:80) yielded pure abiesonic acid (**6**, 10 mg). Fraction A.3 was separated on preparative HPLC (H_2O/MeOH/HCOOH 15:85:0.1) giving four

compounds: (24*E*)-23-oxo-3,4-*seco*-9β*H*-lanosta-4(28),6,8(14),24-tetraen-3,26-dioic acid (**2**, 11 mg), (24*E*)-23-oxo-3,4-*seco*-9β*H*-lanosta- 4(28),7,24-triene-3,26-dioic acid (**3**, 10 mg), firmanoic acid (**7**, 8.7 mg) and 15-hydroxydehydroabietic acid (**15**, 2.5 mg).

In a second purification protocol, oleoresin (1.36 kg, 2nd lot) was triturated in cold hexanes (ratio: 40 g/L of solvent) and the precipitate was filtered off by vacuum filtration on Whatman 4 to give a yellowish powder (Fr B, 45.7 g). The filtrate was evaporated under reduced pressure to give a yellow oil (Fr C, 1.28 kg). The precipitate was subjected to a silica gel column with CH_2Cl_2/MeOH (4:1) to remove nonpolar compounds and washed with MeOH. The washing was purified with CH_2Cl_2/MeOH (25:1 → 15:1) on silica gel column and finally with MeOH to give four fractions Fr B.1–Fr B.4. Fraction B.2 (11.0 g) was purified by silica gel column with CH_2Cl_2/MeOH (50:1 → 30:1 → 20:1 → MeOH) and MeOH fraction was further fractionated by low-pressure liquid chromatography on RP18 silica gel column with a gradient from 70% to 100% MeOH in water to afford four subfractions named B.2.1–B.2.4. Fraction B.2.2 (577 mg) was purified by preparative HPLC (H_2O/MeOH + 0.1% HCOOH 90% → 100%) to obtained four fractions B.2.2.1–Fr B.2.2.4. Fraction B.2.2.2 (174 mg) was separate by preparative HPLC (H_2O/MeOH + 0.1% HCOOH 82% → 85%) and (H_2O/ACN + 0.1% HCOOH 60% → 70%) giving 3α-hydroxy-23-oxocycloart-25(27)-en-26-oic acid (**4**, 14.0 mg) and (25*R*)-3,4-*seco*-9β*H*-lanosta-4(28),7-diene-3,26-dioic acid (**9**, 9.2 mg). Purification of B.2.2.3 (148 mg) by preparative HPLC (H_2O/MeOH + 0.1% HCOOH 88% → 93%) allowed the isolation of awashishinic acid (**5**, 15.2 mg). Preparative HPLC of fraction B.2.3 (636 mg) using (H_2O/ACN + 0.1% HCOOH 87% → 100%) followed by (H_2O/MeOH + 0.1% HCOOH 89% → 92%) led to the isolation of (24*E*)-3,4-*seco*-9β*H*-lanosta-4(28),7,24-triene-3,26-dioic acid (**12**, 6.7 mg).

Filtrate obtained after trituration of oleoresin was also subjected to purification. A part of this filtrate (100.2 g) was treated by liquid/liquid extraction with petroleum ether and NaOH 2% in order to separate

acidic (21.4 g) and neutral (35.6 g) compounds. An aliquot of the neutral fraction (30.0 g) was chromatographed on a silica gel column with hexanes/EtOAc (35:1 → 15:1) and washed with MeOH to give four fractions C.1–C.4. Fraction C.4 (2.8 g) was further purified with a silica gel column according to a gradient of petroleum ether/EtOAc (25:1 → 15:1 → 10:1 → 1:1) and washed with MeOH to obtain eight fractions C.4.1–C.4.8. Fraction C.4.6 (294 mg) was separated on preparative HPLC (H_2O/ACN 70% → 87%) giving methyl 15-hydroxydehydroabietate (**16**, 22.1 mg). Purification by preparative HPLC of fraction C.4.7 using (H_2O/MeOH 60% → 96%) allowed the isolation of methyl 13-oxo-podocarp-8(14)-en-15-oate (**14**, 3.2 mg) and (12*E*)-8-hydroxy-15-nor-12-labden-14-al (**17**, 12.6 mg). Fraction C.4.8 was chromatographed on silica gel column with CH_2Cl_2/MeOH (75:1 → 50:1 → 35:1 → 20:1 → MeOH), with low-pressure liquid chromatography on silica gel column with a gradient from 9% to 100% MeOH in CH_2Cl_2 and finally purified by preparative HPLC (H_2O/ACN 55% → 70%) to obtain abiesanordine C (**13**, 5.0 mg) and 8-hydroxy-14,15-dinor-11-labden-13-one (**18**, 3.1 mg).

Characterization

Abiesonic acid 3-methyl ester (**1**): white amorphous solid; $[\alpha]^{20}_{D}$ −8.8 (*c* 1.0, $CHCl_3$); IR (film) ν_{max} 2961, 2878, 1736, 1692, 1436, 1379, 1272, 1216, 1197, 756 cm^{-1}; ^1H and ^{13}C NMR spectroscopic data, see Tables 1 and 2; HRESIMS *m*/*z* 497.3261 $[M + H]^+$ (calcd for $C_{31}H_{45}O_5$, 497.3262).

(24*E*)-23-Oxo-3,4-*seco*-9β*H*-lanosta-4(28),6,8(14),24-tetraen-3,26-dioic acid (**2**): white amorphous solid; $[\alpha]^{20}_{D}$ −97.5 (*c* 1.0, MeOH); IR (film) ν_{max} 2963, 2878, 1702, 1635, 1372, 1277, 1216, 756 cm^{-1}; ^1H and ^{13}C NMR spectroscopic data, see Tables 1 and 2; HRESIMS *m*/*z* 483.3087 $[M + H]^+$ (calcd for $C_{30}H_{43}O_5$, 483.3105).

(24*E*)-23-Oxo-3,4-*seco*-9β*H*-lanosta-4(28),7,24-triene-3,26-dioic acid (**3**): white amorphous solid; $[\alpha]^{20}_{D}$ −31.5 (*c* 1.0, MeOH); IR (film) ν_{max} 2954, 2880, 1703, 1633, 1377, 1278, 1216, 757 cm^{-1}; ^1H and

^{13}C NMR spectroscopic data, see Tables 1 and 2; HRESIMS *m/z* 485.3250 $[M + H]^+$ (calcd for $C_{30}H_{45}O_5$, 485.3262).

3α-Hydroxy-23-oxocycloart-25(27)-en-26-oic acid (**4**): white amorphous solid; $[\alpha]^{22}_D$ +1.9° (*c* 0.7, CH_2Cl_2:MeOH 1:1); IR (film) ν_{max} 3416, 2930, 2868, 1708, 1633 cm^{-1}; ^{1}H and ^{13}C NMR spectroscopic data, see Tables 1 and 2; HRESIMS *m/z* 471.3463 $[M + H]^+$ (calcd for $C_{30}H_{47}O_4$, 471.3469), *m/z* 493.3282 $[M + Na]^+$ (calcd for $C_{30}H_{46}O_4Na$, 493.3288).

Awashishinic acid (**5**): light brown amorphous solid; $[\alpha]^{22}_D$ +10.7° (*c* 1.5, CH_2Cl_2); IR (film) ν_{max} 3413, 2934, 2863, 1708, 1691, 1622, 1378, 1248 cm^{-1}; ^{1}H and ^{13}C NMR spectroscopic data, see Table S1; HRESIMS *m/z* 471.3465 $[M + H]^+$ (calcd for $C_{30}H_{47}O_4$, 471.3469).

Abiesonic acid (**6**): white amorphous solid; $[\alpha]^{20}_D$ −24.6 (*c* 1.0, MeOH); IR (film) ν_{max} 2963, 2878, 1703, 1381, 1274, 1216, 756 cm^{-1}; ^{13}C and ^{1}H NMR spectroscopic data, see Table S1; HRESIMS *m/z* 483.3093 $[M + H]^+$ (calcd for $C_{30}H_{43}O_5$, 483.3105).

Firmanoic acid (**7**): white amorphous solid; $[\alpha]^{20}_D$ +10.3 (*c* 0.4, $CHCl_3$); IR (film) ν_{max} 2952, 2878, 1698, 1383, 1275, 1247, 1229, 755 cm^{-1}; ^{1}H and ^{13}C NMR, see Table S1; HRESIMS *m/z* 469.3304 $[M + H]^+$ (calcd for $C_{30}H_{45}O_4$, 469.3312).

(22*Z*)-3,4-*seco*-9β*H*-Lanosta-4(28),7,22,24-tetraen-26,23-olid-3-oic acid (**8**): white amorphous solid; $[\alpha]^{20}_D$ −64.3° (*c* 1.5, $CHCl_3$); IR (film) ν_{max} 2954, 1763, 1707, 1452, 1375, 1299, 1215, 1057, 962, 900, 757 cm^{-1}; ^{1}H and ^{13}C NMR, see Table S2; HRESIMS *m/z* 467.3151 $[M + H]^+$ (calcd for $C_{30}H_{43}O_4$, 467.3156).

23-Oxo-3,4-*seco*-9β*H*-lanosta-4(28),7-diene-3,26-dioic acid (**9**): white amorphous solid; $[\alpha]^{22}_{D}$ −26.9° (*c* 0.9, CH_2Cl_2); IR (film) ν_{max} 2951, 2878, 1707, 1635, 1376, 902, 737 cm^{-1}; ^1H and ^{13}C NMR spectroscopic data, see Table S2; HRESIMS *m/z* 487.3414 $[M + H]^+$ (calcd for $C_{30}H_{47}O_5$, 487.3418).

Cytotoxic activity bioassay

The cytotoxic activity bioassay was achieved in a similar manner as described by Mshvildadze et al. [2]. Lung carcinoma (A549), colon adenocarcinoma (DLD-1) and normal skin fibroblast (WS1) human cell lines were purchased from the American Type Culture Collection (ATCC). All cell lines were cultured in minimum essential medium containing Earle's salts and L-glutamine (Mediatech Cellgro, VA), to which were added 10% fetal bovine serum (Hyclone), vitamins (1×), penicillin (100 I.U./mL) and streptomycin (100 μg/mL), essential amino acids (1×) and sodium pyruvate (1×) (Mediatech Cellgro, VA). Cells were kept at 37 °C in a humidified environment containing 5% CO_2. Exponentially growing cells were plated in 96-well microplates (Costar, Corning Inc.) at a density of 5×10^3 cells per well in 100 μL of culture medium and were allowed to adhere for 16 h before treatment. Increasing concentrations of each compound in DMSO (Sigma-Aldrich) were then added (100 μL per well) and the cells were incubated for 48 h. The final concentration of DMSO in the culture medium was maintained at 0.5% (v/v) to avoid solvent toxicity. Cytotoxicity was assessed using resazurin reduction test [3] on an automated 96-well Fluoroskan Ascent F1™ plate reader (Labsystems) using excitation and emission wavelengths of 530 nm and 590 nm, respectively. Fluorescence was proportional to the cellular metabolic activity in each well. Survival percentages were defined as the fluorescence in experimental wells compared to that in control wells after subtraction of blank values. Cytotoxicity results were expressed as mean ± standard deviation and represent the concentration inhibiting 50% of cell growth (IC_{50}). Each experiment was carried out three times in triplicate.

Antibacterial bioassay

Antibacterial activity was evaluated using the microdilution method [4] with some modifications. Briefly, exponentially growing bacteria were plated in 96-well flat-bottom microplates (BD Falcon) at a density of 5×10^3 Gram-negative *E. coli* (ATCC 25922) or 40×10^3 Gram-positive *S. aureus* (ATCC 25923) per well in 100 μL nutrient broth (Difco). Increasing concentrations of compounds in DMSO (Sigma-Aldrich) were then added (100 μL per well). The final concentration of DMSO in the culture medium was maintained at 0.25% (v/v) to avoid solvent toxicity. Fifty microliters of 4% resazurin was added to each well and the microplates were incubated for 6 h at 37 °C. Absorbance was measured after 6 h on an automated 96-well Varioskan plate reader (Thermo-Labsystems) at wavelengths of 600 nm and 660 nm for *E. coli* and *S. aureus* respectively.

Complete NMR assignment for known compounds

Table S1: NMR Spectroscopic data (400 MHz) for compounds **5**–**7**.

	**5**[a]		**6**[b]		**7**[c]	
Position	δ_C, mult	δ_H, mult (*J* in Hz)	δ_C, mult	δ_H, mult (*J* in Hz)	δ_C, mult	δ_H, mult (*J* in Hz)
1	28.1, CH_2	2.32, m<br>1.06, m	30.3, CH_2	1.76, m<br>1.66, m	35.2, CH_2	1.78, m<br>1.66, m
2	29.7, CH_2	2.04, m<br>1.90, m	29.1, CH_2	2.36, m	35.4, CH_2	2.55, m<br>2.49, m
3	76.1, CH	3.72, br s	180.3, C	-	221.8, C	-
4	40.1, C	-	149.1, C	-	48.1, C	-
5	41.1, CH	2.28, m	44.1, CH	2.09, m	53.8, CH	1.48, m
6	21.4, CH_2	1.54, m<br>0.76, m	30.9, CH_2	2.41, m<br>2.15, m	24.0, CH_2	1.95, m<br>1.85, m
7	26.1, CH_2	1.29, m<br>1.10, m	122.4, CH	5.49, dd (6.2, 3.1)	122.9, CH	5.69, dt (7.8, 2.5)
8	48.2, CH	1.53, m	143.4, C	-	149.9, C	-
9	19.9, C	-	49.5, CH	2.08, m	46.8, CH	2.28, m
10	27.1, C	-	36.9, C	-	37.0, C	-
11	26.5, CH_2	2.02, m<br>1.16, m	22.5, CH_2	1.61, m<br>1.41, m	21.9, CH_2	1.68, m<br>1.11, m
12	33.1, CH_2	1.59, m	31.2, CH_2	1.78, m<br>1.33, m	35.5, CH_2	1.90, m<br>1.71, m
13	45.6, C	-	63.5, C	-	45.3, C	-
14	49.3, C	-	161.0, C	-	53.2, C	-
15	35.7, CH_2	1.31, m	27.8, CH_2	2.47, m<br>2.37, m	34.3, CH_2	1.66, m<br>1.47, m
16	28.6, CH_2	1.89, m<br>1.29, m	36.1, CH	1.57, m	29.5, CH_2	2.01, m<br>1.31, m
17	52.6, CH	1.65, m	50.3, C	-	54.4, CH	1.63, m
18	18.3, CH_3	1.03, o	17.7, CH_3	0.90, s	22.9, CH_3	0.86, s
19	29.9, CH_2	0.50, d (4.1)<br>0.34, d (4.1)	24.7, CH_3	0.93, s	23.5, CH_3	1.00, s
20	33.5, CH	2.24, m	33.8, CH	2.38, m	34.7, CH	2.04, m
21	19.7, CH_3	1.03, o	16.4, CH_3	0.85, d (6.4)	20.0, CH_3	0.91, d (6.4)
22	52.3, CH_2	2.74, br d (14.0)<br>2.38, dd (15.4, 9.6)	48.3, CH_2	2.51, m<br>2.25, m	52.8, CH_2	2.68, dd (15.6, 3.0)<br>2.28, m
23	202.8, C	-	202.3, C	-	204.7, C	-
24	132.5, CH	7.60, m	135.0, CH	7.13, q-like (1.5)	133.0, CH	7.07, br d (0.9)
25	143.1, C	-	138.4, C	-	143.6, C	-
26	170.9, C	-	172.7, C	-	171.6, C	-
27	15.2, CH_3	2.61, s	13.9, CH_3	2.18, d (1.5)	14.9, CH_3	2.15, d (1.3)
28	26.9, CH_3	1.23, s	112.0, CH_2	4.87, s<br>4.79, d (2.5)	28.5, CH_3	1.08, s
29	21.7, CH_3	0.98, s	26.2, CH_3	1.76, s	21.7, CH_3	1.09, s
30	19.5, CH_3	0.91, s	106.9, CH_2	4.77, s<br>4.72, s	27.9, CH_3	1.06, s

[a]Acquired in pyridine-d_5; [b]acquired in $CDCl_3$; [c]acquired in CD_3OD.

Table S2: NMR Spectroscopic data (400 MHz, $CDCl_3$) for compounds **8** and **9**.

	**8**		**8**[a]		**9**	
Position	δ_C, mult	δ_H, mult (*J* in Hz)	δ_C, mult	δ_H, mult (*J* in Hz)	δ_C, mult	δ_H, mult (*J* in Hz)
1	28.8, CH_2	1.72, m	28.7, CH_2	1.71, m	28.8, CH_2	1.71, m
		1.59, m		1.60, m		1.59, m
2	29.1, CH_2	2.31, m	29.1, CH_2	2.31, m	29.3, CH_2	2.29, m
3	180.3, C	-	180.2, C	-	180.7, C	-
4	149.6, C	-	149.6, C	-	149.7, C	-
5	45.3, CH	2.08, m	45.3, CH	2.08, d (6.9)	45.3, CH	2.07, d (6.7)
6	29.7, CH_2	2.28, m	29.6, CH_2	2.25, m	29.7, CH_2	2.27, m
		1.98, m		1.99, m		1.98, m
7	118.1, CH	5.31, br s	118.1, CH	5.32, br s	118.0, CH	5.32, br s
8	146.2, C	-	146.2, C	-	146.4, C	-
9	38.6, CH	2.60, m	38.6, CH	2.60, d (10.6)	38.6, CH	2.57, m
10	36.3, C	-	36.3, C	-	36.3, C	-
11	18.5, CH_2	1.60, m	18.4, CH_2	1.68, m	18.5, CH_2	1.61, m
				1.58, m		
12	33.7, CH_2	1.87, m	33.7, CH_2	1.86, m	33.8, CH_2	1.82, m
		1.69, m		1.68, m		1.66, m
13	44.0, C	-	44.0, C	-	43.8, C	-
14	51.6, C	-	51.6, C	-	51.6, C	-
15	34.1, CH_2	1.52, m	34.1, CH_2	1.51, m	34.0, CH_2	1.51, m
		1.42, m		1.41, m		
16	27.9, CH_2	1.72, m	27.9, CH_2	1.74, m	28.4, CH_2	1.90, m
		1.27, m		1.25, m		1.25, m
17	53.0, CH	1.64, m	53.0, CH	1.65, m	53.0, CH	1.51, m
18	21.9, CH_3	0.81, s	21.9, CH_3	0.82, s	21.7, CH_3	0.78, s
19	24.1, CH_3	0.86, br s	24.1, CH_3	0.86, s	24.1, CH_3	0.85, s
20	34.4, CH	2.88, m	34.4, CH	2.89, m	33.0, CH	1.99, m
21	19.9, CH_3	1.05, d (6.9)	19.8, CH_3	1.05, d (6.6)	19.3, CH_3	0.86, d (8.1)
22	121.0, CH	4.98, d (10.3)	121.0, CH	4.98, d (10.4)	50.0, CH_2	2.46, br d (15.8)
						2.19, dd (15.8, 10.2)
23	146.5, C	-	146.5, C	-	209.4, C	-
24	138.1, CH	6.97, d (1.3)	138.1, CH	6.97, br s	46.4, CH_2	2.82, dd (17.5, 9.2)
						2.49, dd (17.6, 4.6)
25	128.8, C	-	128.8, C	-	34.7, CH	2.97, m
26	171.4, C	-	171.4, C	-	181.8, C	-
27	10.5, CH_3	1.99, br s	10.5, CH_3	1.99, s	16.9, CH_3	1.22, d (7.1)
28	112.1, CH_2	4.89, s	112.1, CH_2	4.89, s	112.0, CH_2	4.88, s
		4.82, s		4.82, s		4.82, s
29	26.0, CH_3	1.80, m	25.9, CH_3	1.80, s	26.0, CH_3	1.79, s
30	27.5, CH_3	1.03, m	27.4, CH_3	1.04, s	27.5, CH_3	1.03, s

[a]From Xia et al. [5].

Table S3: NMR Spectroscopic data (400 MHz) for compounds **10–12**.

	**10**[a]		**11**[a]		**12**[b]	
Position	δ_C, mult	δ_H, mult (*J* in Hz)	δ_C, mult	δ_H, mult (*J* in Hz)	δ_C, mult	δ_H, mult (*J* in Hz)
1	28.8, CH_2	1.71, m<br>1.58, m	28.5, CH_2	1.59, m	29.7, CH_2	2.26, m
2	29.1, CH_2	2.31, m	29.9, CH_2	2.29, m	178.1, C	-
3	180.1, C	-	180.0, C	-	150.5, C	-
4	149.6, C	-	145.9, C	-	46.0, CH	2.12, m
5	45.3, CH	2.08, m	50.6, CH	2.62, d (5.1)	30.2, CH_2	2.32, m<br>1.99, m
6	29.6, CH_2	2.27, m<br>1.99, m	126.7, CH	5.36, dd (9.8, 5.5)	118.5, CH	5.34, m
7	118.0, CH	5.32, br s	125.3, CH	6.21, d (9.9)	147.3, C	-
8	146.3, C	-	125.1, C	-	39.5, CH	2.61, m
9	38.7, CH	2.58, m	39.5, CH	2.39, m	36.9, C	-
10	36.3, C	-	37.1, C	-	19.2, CH_2	1.68, m<br>1.58, m
11	18.6, CH_2	1.64, m<br>1.55, m	23.9, CH_2	2.38, m<br>2.29, m	34.7, CH_2	1.87, m<br>1.70, m
12	34.0, CH_2	1.83, m<br>1.68, m	32.5, CH_2	1.60, m	44.3, C	-
13	43.8, C	-	47.5, C	-	52.2, C	-
14	51.6, C	-	146.7, C	-	34.6, CH_2	1.53, m<br>1.49, m
15	34.0, CH_2	1.53, m<br>1.45, m	19.8, CH_2	1.57, m	28.8, CH_2	1.98, m<br>1.30, m
16	28.3, CH_2	1.91, m<br>1.27, m	36.2, CH_2	1.71, m<br>1.50, m	53.6, CH	1.53, m
17	53.5, CH	1.46, m	49.1, C	-	22.1, CH_3	0.79, s
18	21.7, CH_3	0.75, s	15.6, CH_3	0.65, s	24.4, CH_3	0.88, s
19	24.1, CH_3	0.85, s	21.9, CH_3	0.85, s	36.7, CH	1.44, m
20	33.1, CH	1.70, m	34.4, CH	2.08, m	18.5, CH_3	0.94, d (6.3)
21	18.2, CH_3	0.95, d (6.4)	15.3, CH_3	0.93, d (6.7)	35.3, CH_2	1.59, m<br>1.19, m
22	42.6, CH_2	1.79, m<br>1.18, m	38.7, CH_2	1.91, m<br>1.21, m	26.3, CH_2	2.26, m<br>2.13, m
23	76.0, CH	4.66, m	76.7, CH	4.62, m	144.3, CH	6.81, t (7.4)
24	36.4, CH_2	2.05, m	36.5, CH_2	2.06, m	127.9, C	-
25	34.2, CH	2.71, m	34.1, CH	2.71, m	171.4, C	-
26	180.3, C		180.0, C	-	12.4, CH_3	1.83, s
27	15.9, CH_3	1.29, d (7.5)	15.9, CH_3	1.29, d (7.3)	112.2, CH_2	4.88, br s<br>4.84, br s
28	112.0, CH_2	4.88, br s<br>4.82, s	115.4, CH_2	4.96, s<br>4.75, s	26.2, CH_3	1.81, s
29	26.0, CH_3	1.79, s	24.8, CH_3	1.78, s	27.8, CH_3	1.06, s
30	27.5, CH_3	1.02, s	21.7, CH_3	1.02, s	29.7, CH_2	2.26, m

[a]Acquired in $CDCl_3$; [b]acquired in $CDCl_3/CD_3OD$ 1:1.

Table S4: NMR Spectroscopic data (400 MHz, $CDCl_3$) for compounds **13** and **14**.

	**13**		**14**	
Position	δ_C, mult	δ_H, mult (*J* in Hz)	δ_C, mult	δ_H, mult (*J* in Hz)
1	38.2, CH_2	1.84, m		1.79, m
		1.17, m	38.3, CH_2	1.22, m
2	17.1, CH_2	1.65, m	17.9, CH_2	1.59, m
3	32.4, CH_2	1.41, m		1.74, m
			36.7, CH_2	1.64, m
4	49.5, C	-	47.3, C	-
5	45.8, CH	1.75, m	48.2, CH	2.07, dd (12.6, 2.6)
6	23.7, CH_2	1.58, m		1.59, m
		1.26, m	24.1, CH_2	1.34, m
7	35.0, CH_2	2.51, dd (15.0, 3.8)		2.50, ddd (15.8, 4.9, 1.5)
		2.35, m	35.2, CH_2	2.35, m
8	164.2, C	-	164.9, C	-
9	51.3, CH	2.19, m	51.6, CH	2.19, m
10	37.8, C	-	38.4, C	-
11	20.4, CH_2	2.04, m		2.02, m
		1.77, m	20.4, CH_2	1.75, m
12	36.7, CH_2	2.42, m		2.41, dt (15.8, 4.2)
		2.24, m	36.8, CH_2	2.25, m
13	199.6, C	-	199.8, C	-
14	126.4, CH	5.90, s	126.2, CH	5.88, br s
15	-	-	-	-
16	-	-	-	-
17	-	-	-	-
18	205.8, CH	9.28, s	179.0, C	-
19	14.8, CH_3	1.14, s	17.0, CH_3	1.23, s
20	15.8, CH_3	0.88, s	15.6, CH_3	0.85, s
OMe			52.1, CH_3	3.69, s

Table S5: NMR Spectroscopic Data (400 MHz, $CDCl_3$) for compounds **15** and **16**.

	**15**		**16**	
Position	δ_C, mult	δ_H, mult (*J* in Hz)	δ_C, mult	δ_H, mult (*J* in Hz)
1		2.32, m		2.31, br d (12.3)
	37.9, CH_2	1.48, m	37.9, CH_2	1.49, m
2	18.5, CH_2	1.77, m	18.5, CH_2	1.74, m
3		1.80, m		1.76, m
	36.7, CH_2	1.72, m	36.6, CH_2	1.64, m
4	47.3, C	-	47.6, C	-
5	44.6, CH	2.24, m	44.8, CH	2.23, dd (12.5, 1.9)
6		1.87, m		1.83, m
	21.7, CH_2	1.56, m	21.7, CH_2	1.41, m
7	30.1, CH_2	2.93, m	30.1, CH_2	2.90, m
8	134.7, C	-	134.7, C	-
9	147.8, C	-	147.9, C	-
10	36.9, C	-	37.0, C	-
11	124.1, CH	7.22, m	124.1, CH	7.22, m
12	122.0, CH	7.22, m	122.0, CH	7.23, m
13	146.0, C	-	146.0, C	-
14	124.9, CH	7.16, m	124.9, CH	7.15, br s
15	72.3, C	-	72.3, C	-
16	31.6, CH_3	1.56, s	31.6, CH_3	1.56, m
17	31.6, CH_3	1.56, s	31.6, CH_3	1.56, m
18	183.7, C	-	179.1, C	-
19	16.3, CH_3	1.29, s	16.5, CH_3	1.28, m
20	25.1, CH_3	1.22, s	25.1, CH_3	1.21, m
OMe	-	-	52.0, CH_3	3.66, s

Table S6: NMR Spectroscopic data (400 MHz) for compounds **17** and **18**.

	**17**[a]		**18**[b]	
Position	δ_C, mult	δ_H, mult (*J* in Hz)	δ_C, mult	δ_H, mult (*J* in Hz)
1	41.6, CH_2	1.55, m	41.0, CH_2	1.34, m
		0.94, m		0.88, m
2	19.5, CH_2	1.65, m	18.3, CH_2	1.58, m
		1.41, m		1.40, m
3	43.0, CH_2	1.38, m	41.8, CH_2	1.40, m
		1.18, m		1.15, m
4	34.2, C	-	33.3, C	-
5	57.5, CH	1.01, m	55.5, CH	0.93, dd (12.1, 2.1)
6	21.5, CH_2	1.70, m	20.2, CH_2	1.71, m
		1.37, m		1.34, m
7	45.2, CH_2	1.87, dt (12.3, 3.1)	42.9, CH_2	1.93, dt (12.5, 3.2)
		1.49, m		1.50, m
8	74.4, C	-	72.4, C	-
9	63.1, CH	1.48, m	65.9, CH	1.95, d (10.6)
10	40.3, C	-	37.9, C	-
11	26.4, CH_2	2.57, dt (9.6, 5.1)	144.6, CH	6.85, dd (15.6, 10.6)
		2.43, ddd (13.7, 8.3, 6.1)		
12	161.9, CH	6.72, t (7.0)	135.4, CH	6.20, d (15.6)
13	138.1, C	-	197.5, C	
14	197.4, CH	9.32, s	-	-
15	-	-	-	-
16	9.3, CH_3	1.74, s	27.8, CH_3	2.28, s
17	23.8, CH_3	1.16, s	25.1, CH_3	1.27, s
18	22.0, CH_3	0.84, s	33.4, CH_3	0.89, s
19	33.9, CH_3	0.89, s	21.6, CH_3	0.82, s
20	16.1, CH	0.92, s	16.0, CH_3	0.99, s

[a]Acquired in CD_3OD; [b]acquired in $CDCl_3$.

References

1. Lavoie, S.; Legault, J.; Gauthier, C.; Mshvildadze, V.; Mercier, S.; Pichette, A. *Org. Lett.* **2012,** *14*, 1504–1507.

2. Mshvildadze, V.; Legault, J.; Lavoie, S.; Gauthier, C.; Pichette, A. *Phytochemistry* **2007,** *68*, 2531–2536.

3. O'Brien, J.; Wilson, I.; Orton, T.; Pognan, F. *Eur. J. Biochem.* **2000,** *267*, 5421–5426.

4. Banfi, E.; Scialino, G.; Monti-Bragadin, C. *J. Antimicrob. Chemother.* **2003,** *52*, 796–800.

5. Xia, J. H.; Zhang, S. D.; Li, Y. L.; Wu, L.; Zhu, Z. J.; Yang, X. W.; Zeng, H. W.; Li, H. L.; Wang, N.; Steinmetz, A.; Zhang, W. D. *Phytochemistry* **2012,** *74*, 178–184.

NMR Spectra of all isolated compounds

^{1}H NMR spectrum of **1** ($CDCl_3$, 400 MHz)

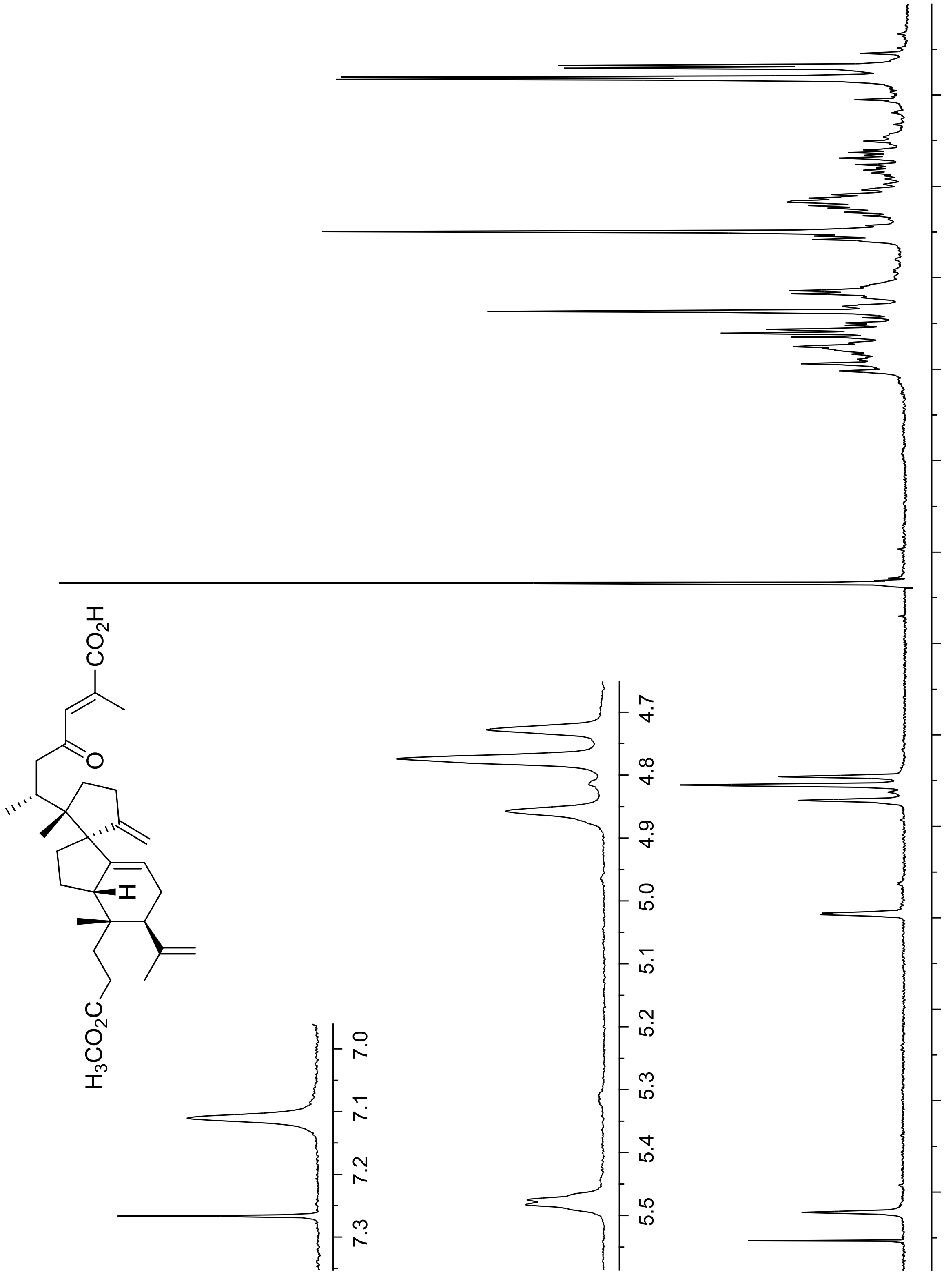

^{13}C NMR spectrum of **1** ($CDCl_3$, 100 MHz)

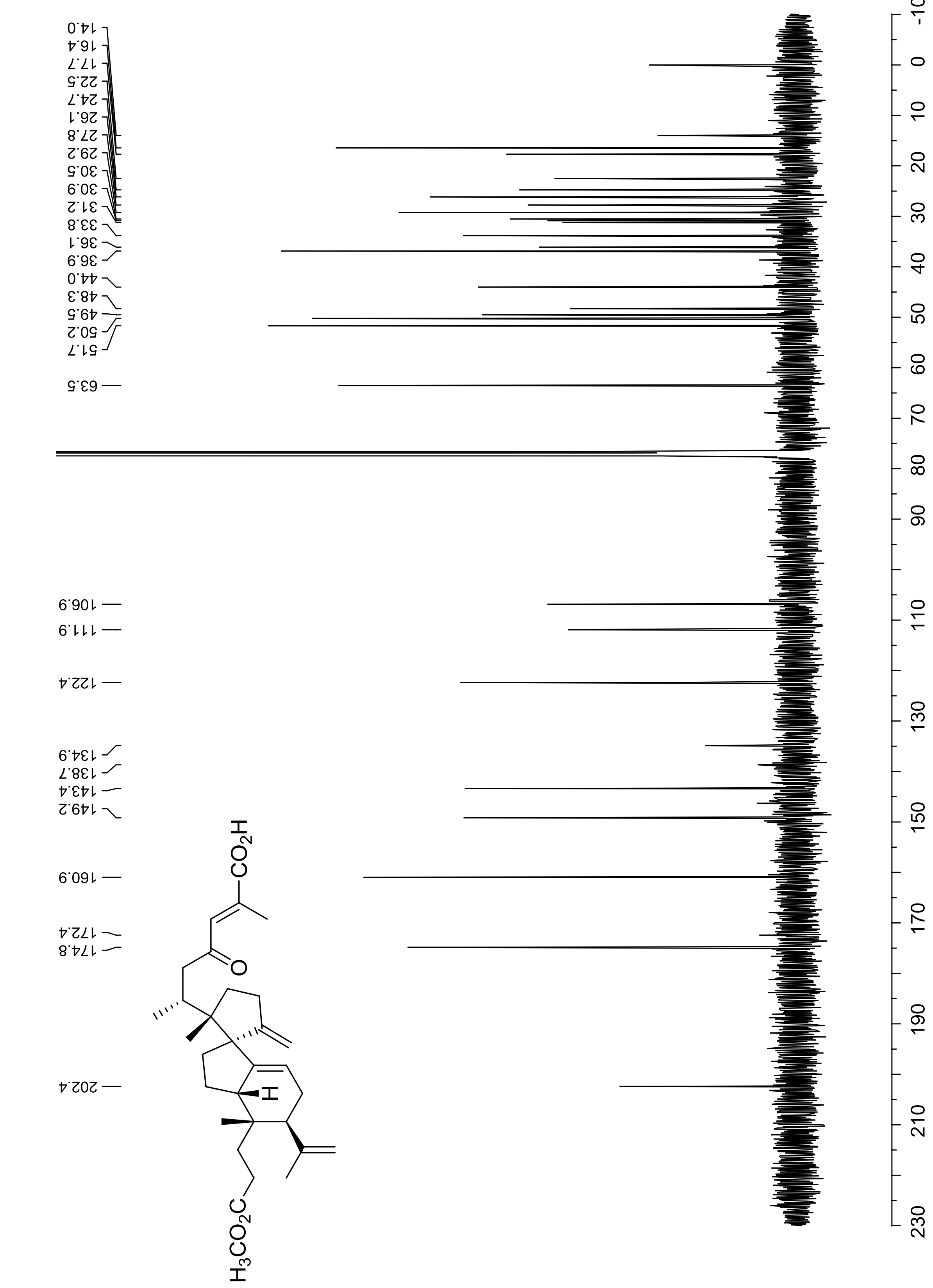

<u>^{1}H NMR spectrum of **2** ($CDCl_3$, 400 MHz)</u>

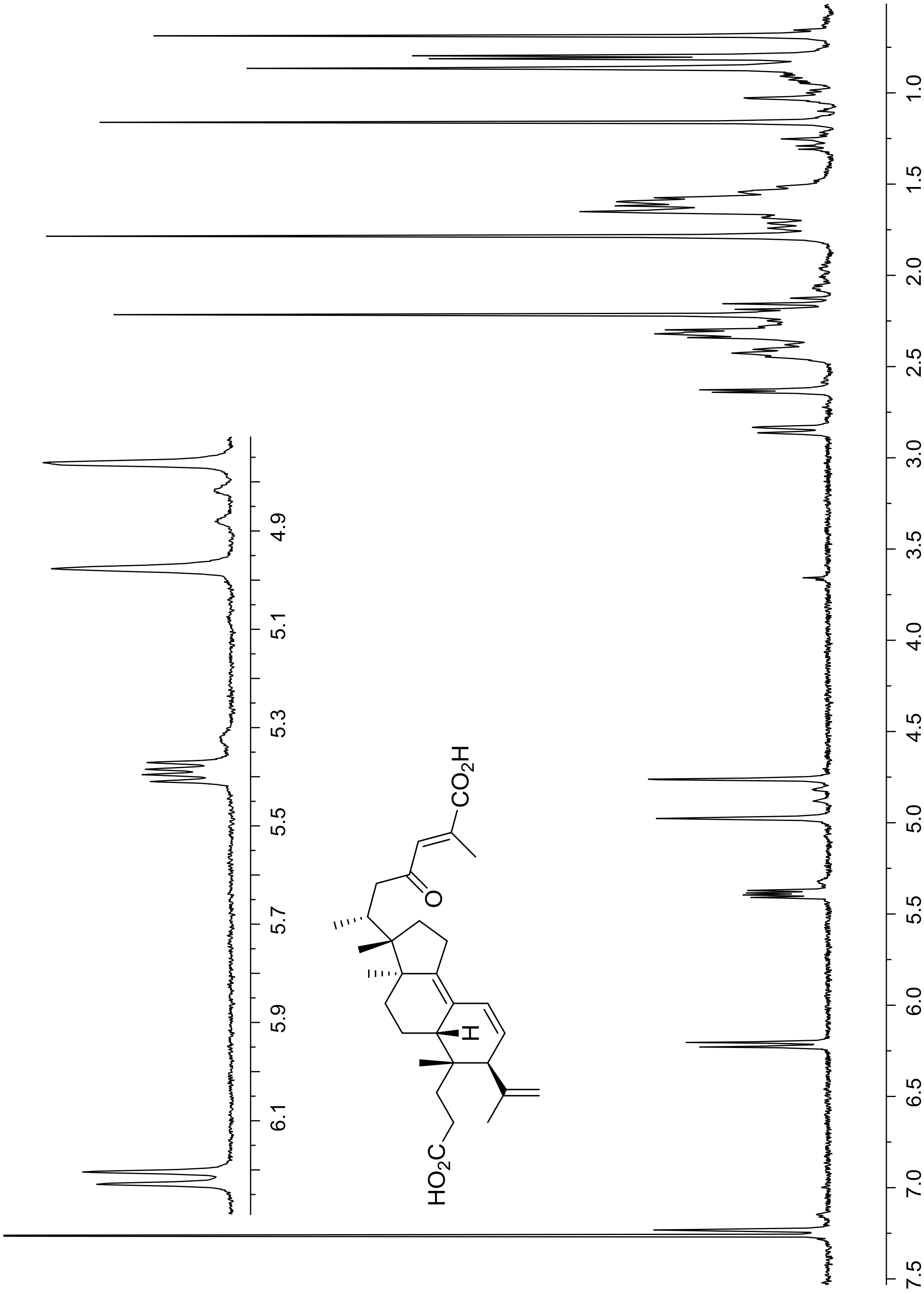

^{13}C NMR spectrum of **2** ($CDCl_3$, 100 MHz)

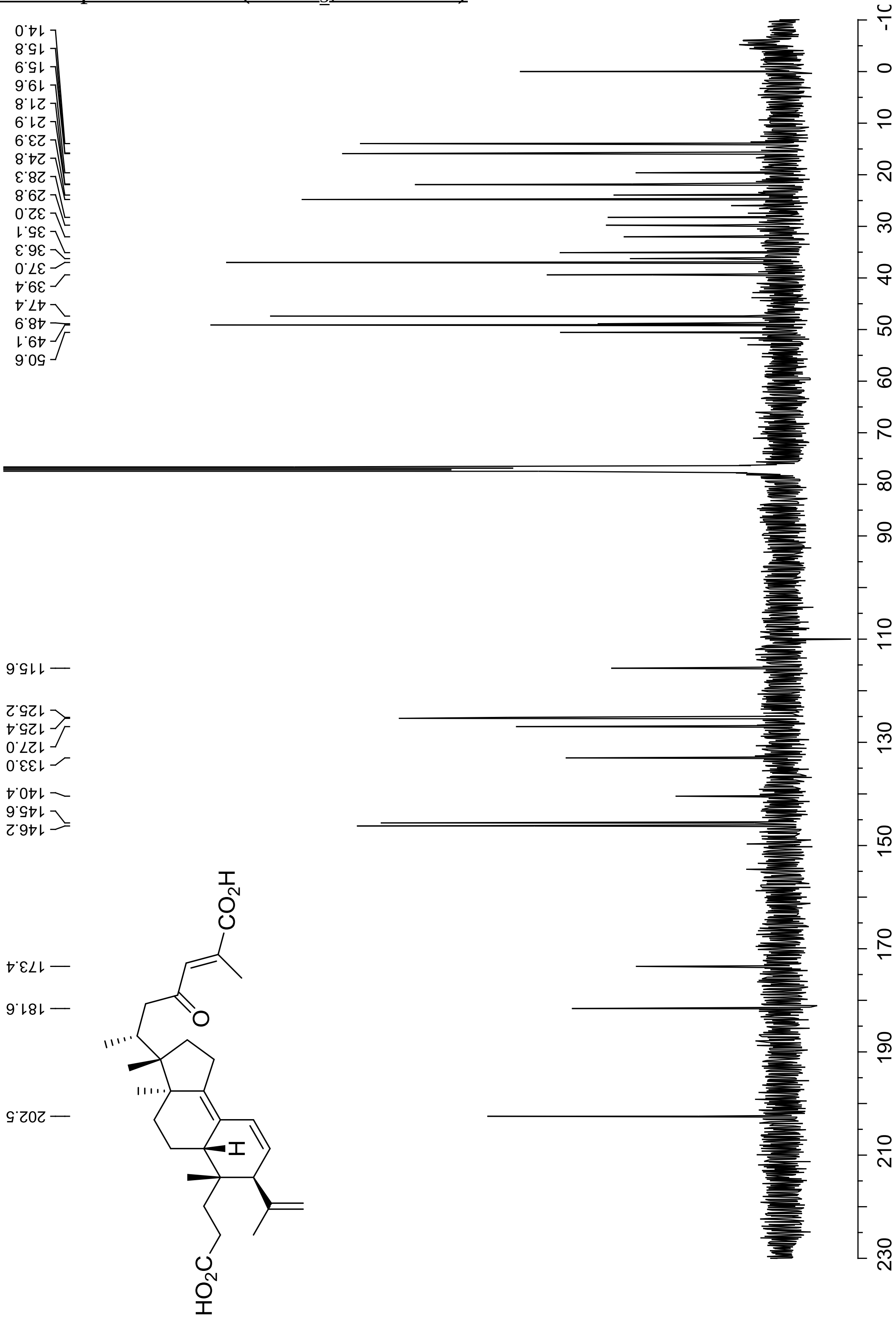

^{1}H NMR spectrum of **3** ($CDCl_3$, 400 MHz)

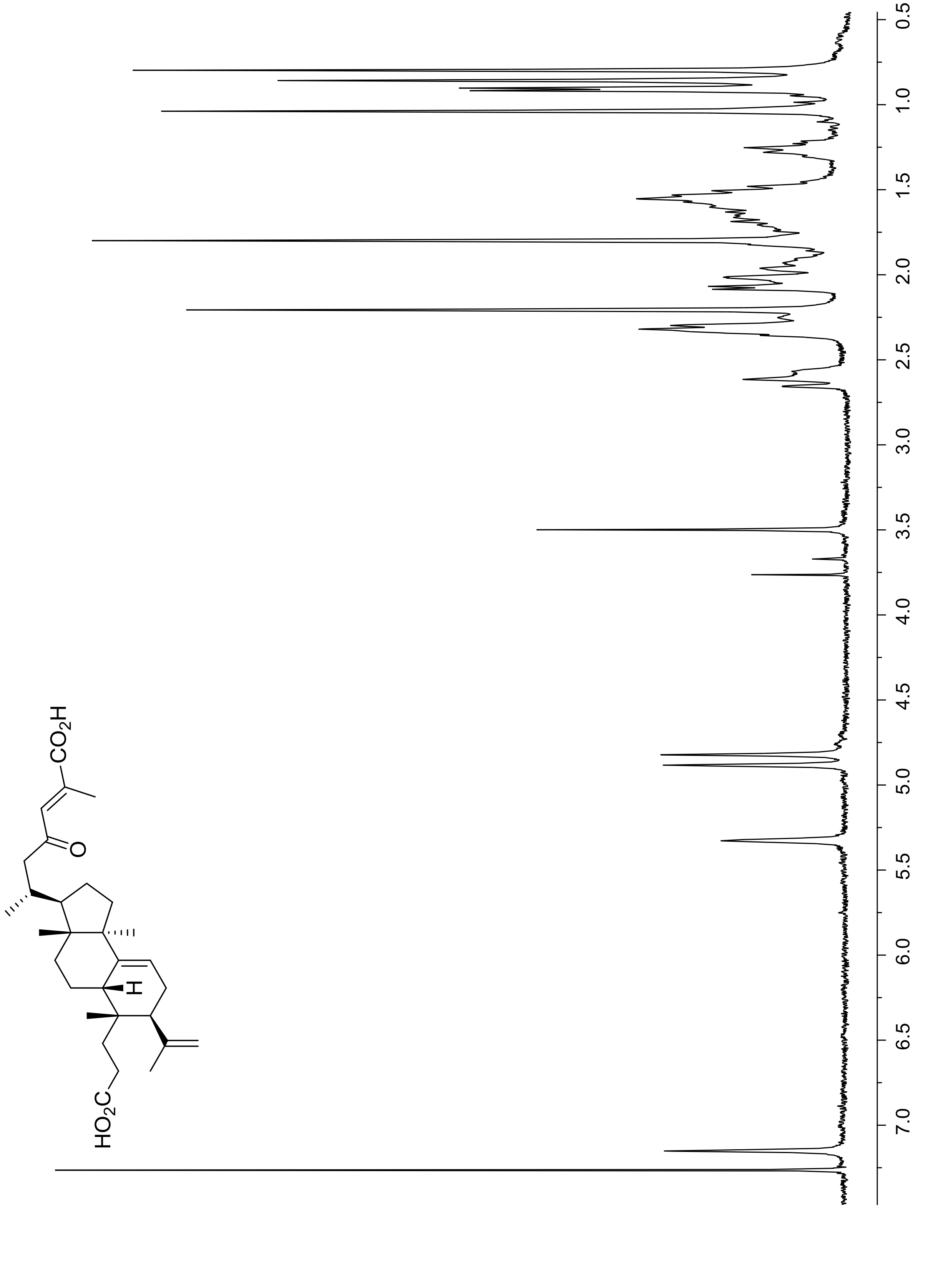

^{13}C NMR spectrum of **3** ($CDCl_3$, 100 MHz)

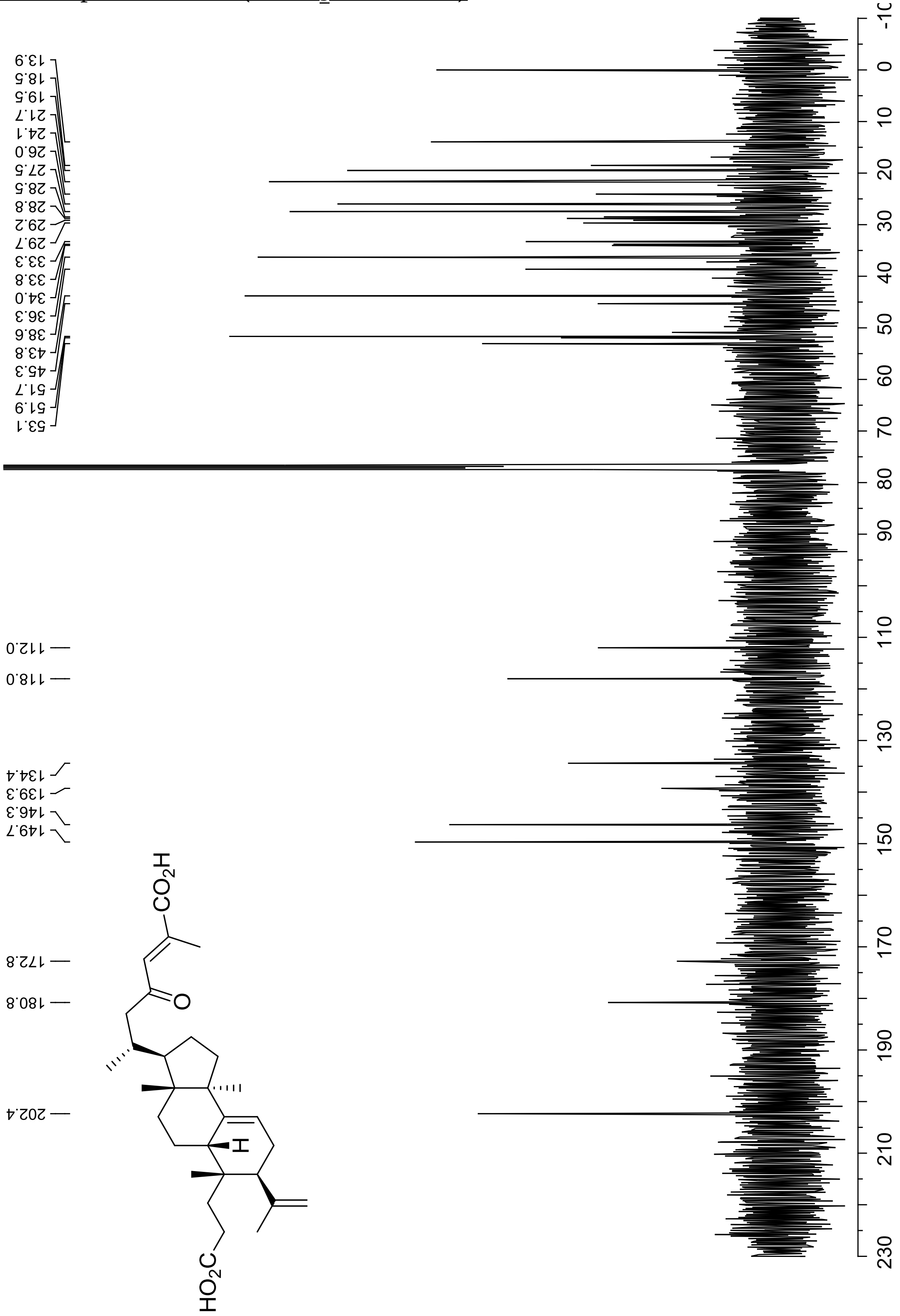

^{1}H NMR spectrum of **4** ($CDCl_3$, 400 MHz)

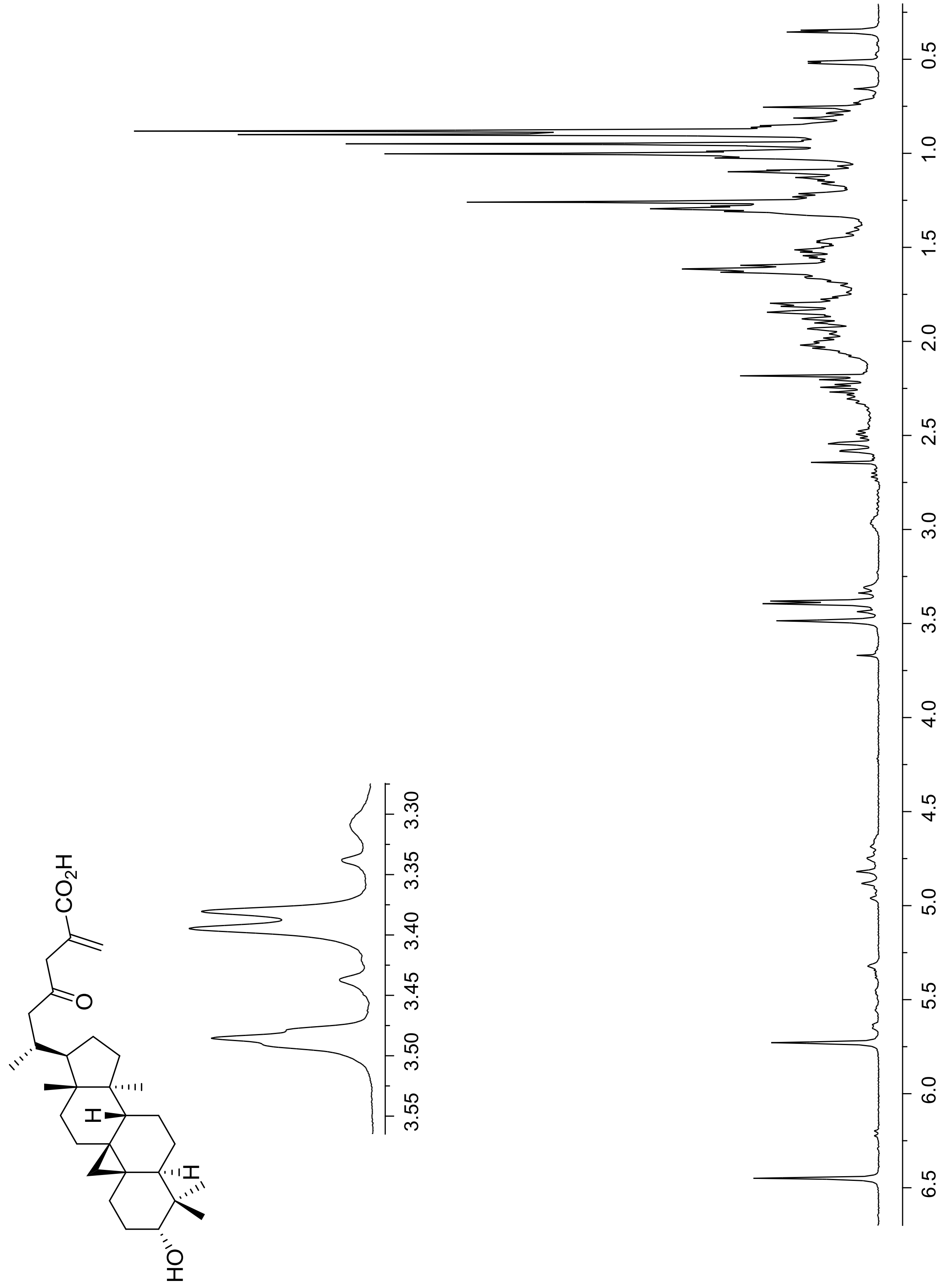

^{13}C NMR spectrum of **4** ($CDCl_3$, 100 MHz)

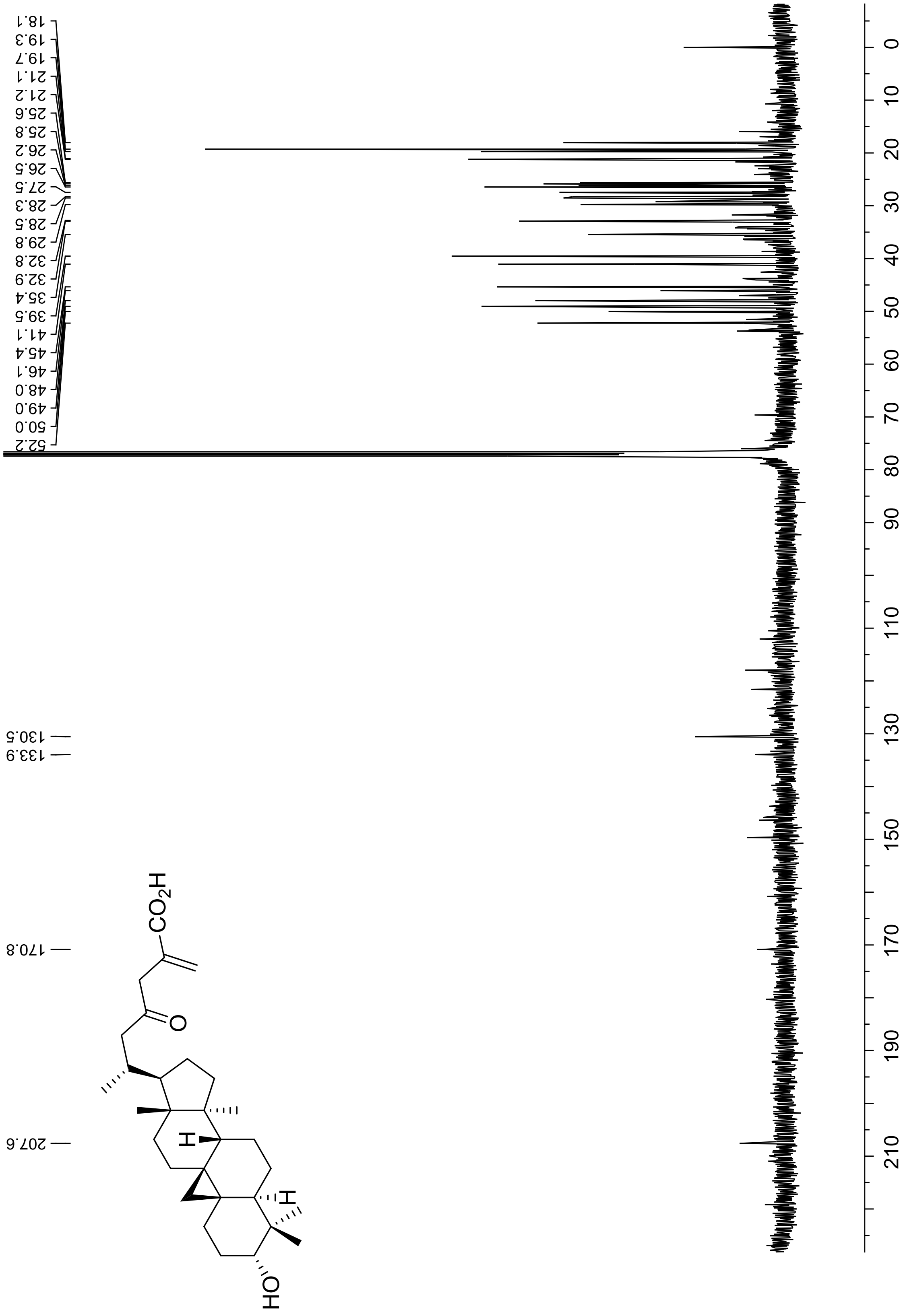

^{1}H NMR spectrum of **5** (pyridine-*d*$_5$, 400 MHz)

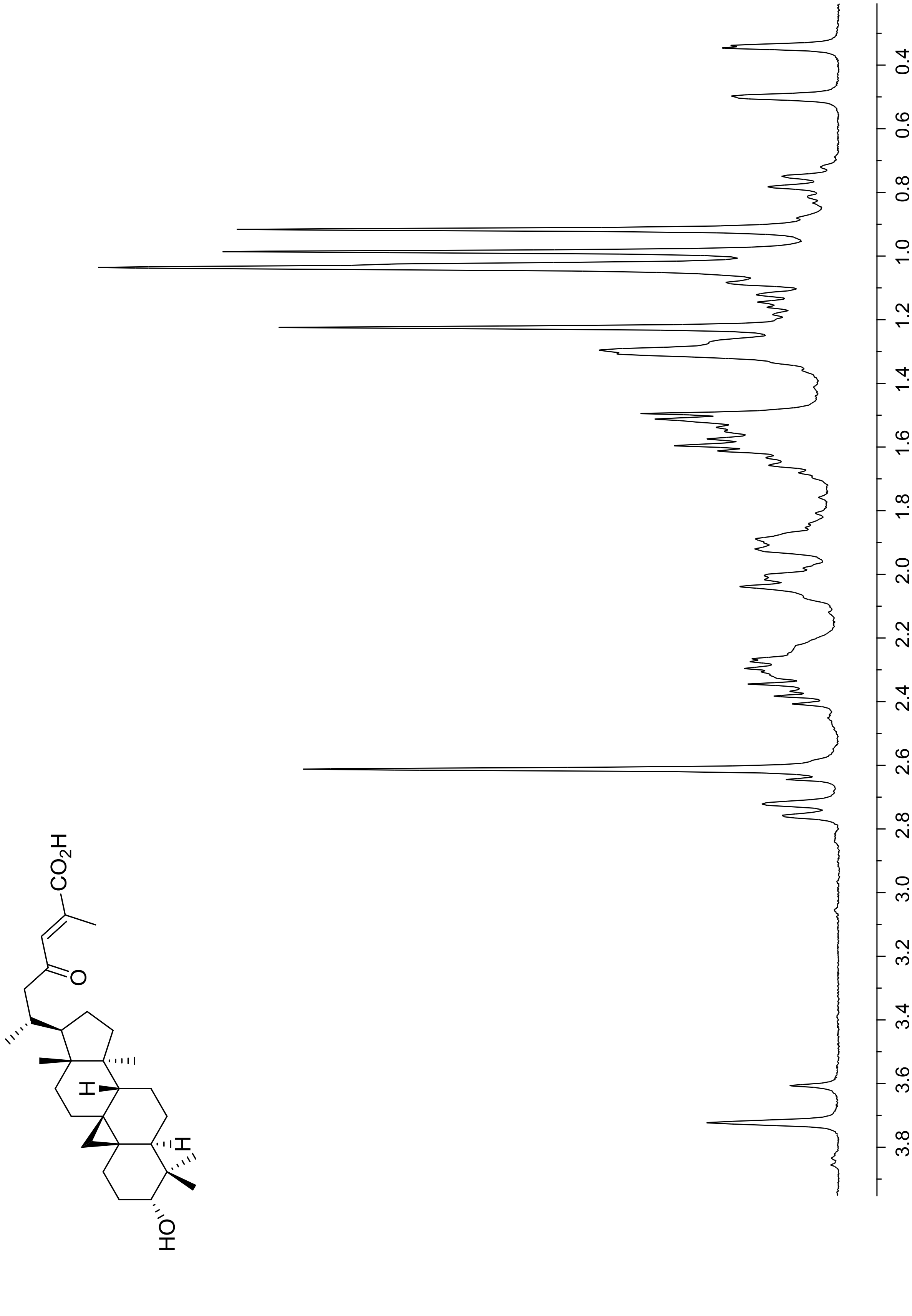

^{13}C NMR spectrum of **5** (pyridine-d_5, 100 MHz)

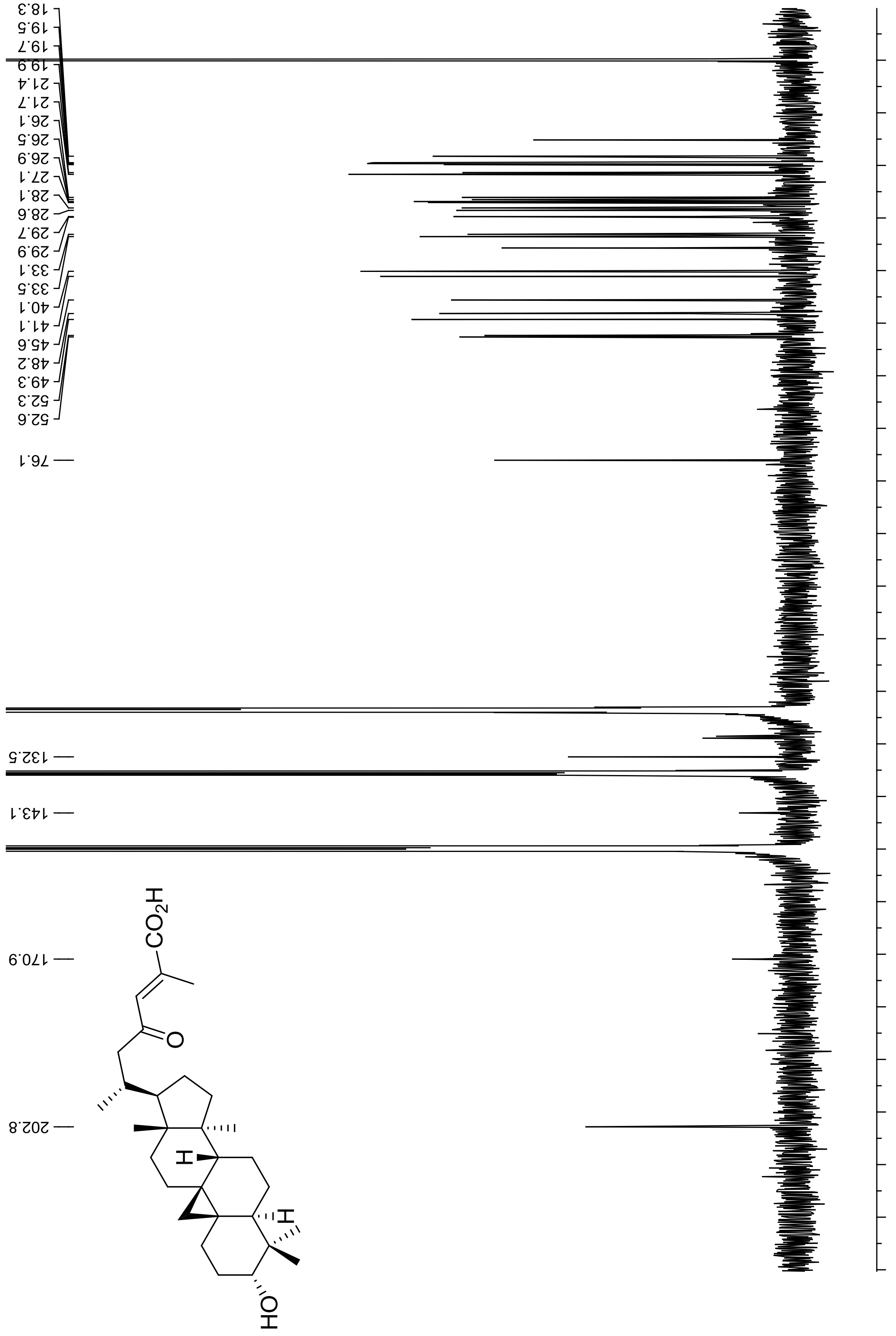

^{1}H NMR spectrum of **6** ($CDCl_3$, 400 MHz)

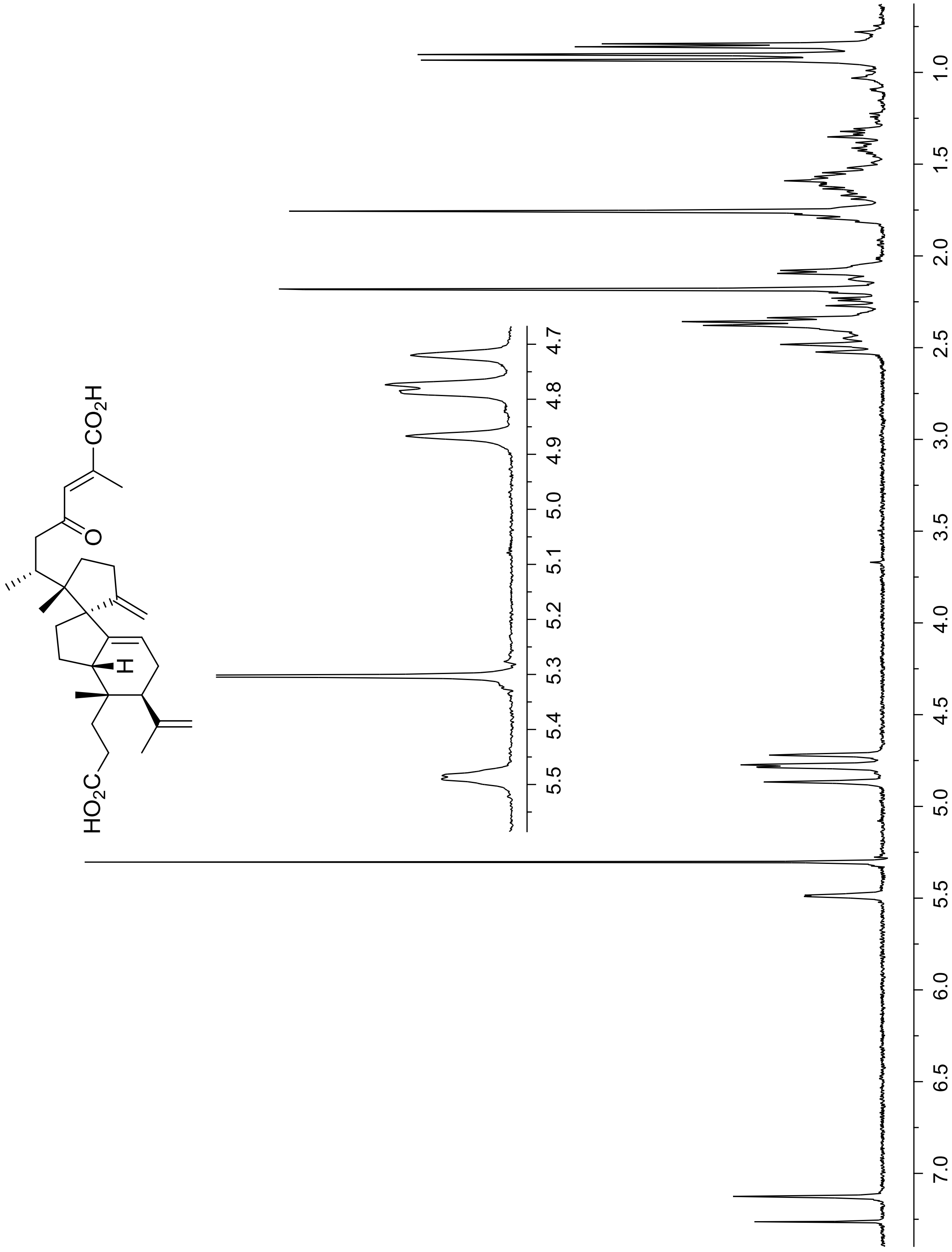

^{13}C NMR spectrum of **6** ($CDCl_3$, 100 MHz)

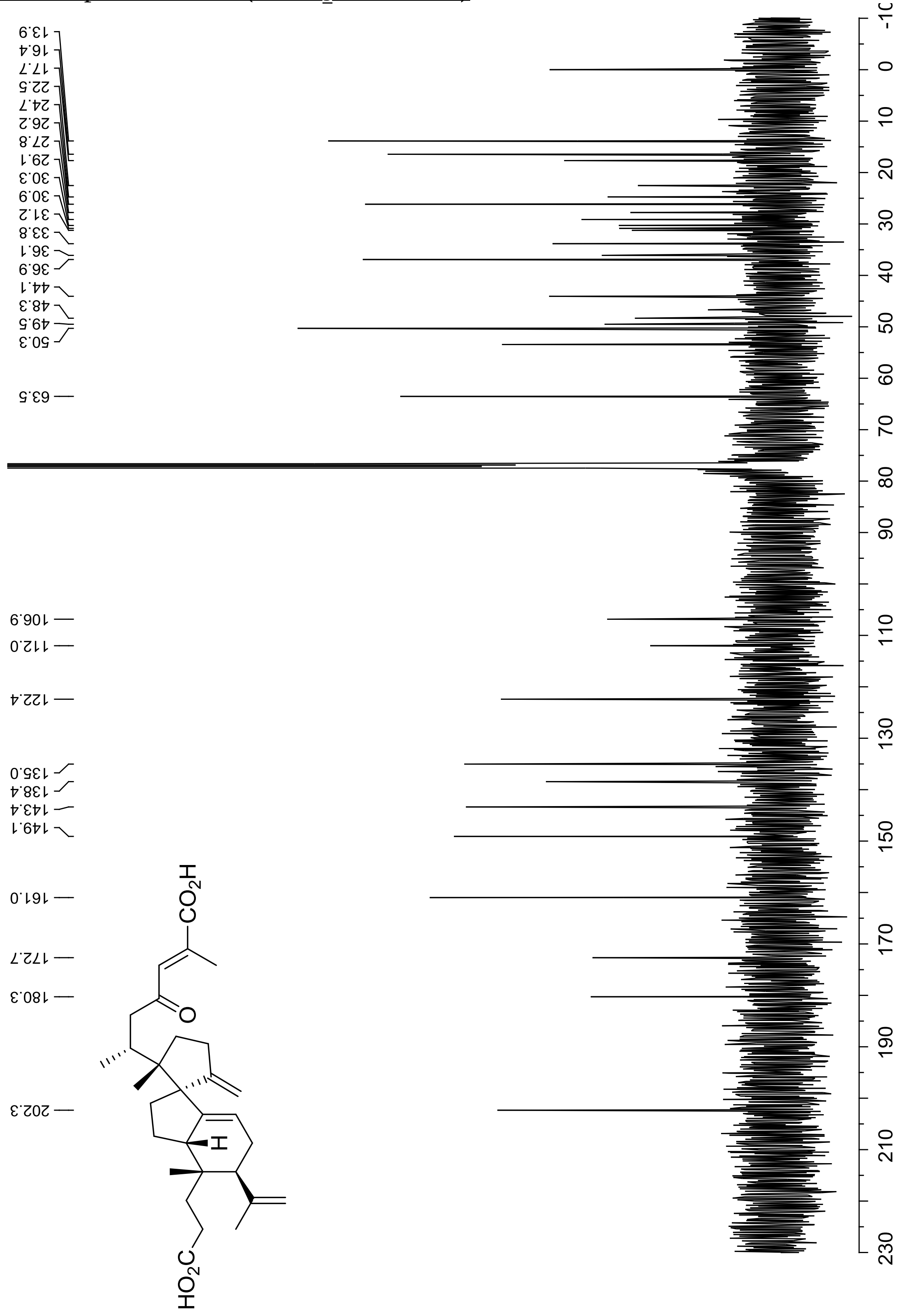

^{1}H NMR spectrum of **7** (CD_3OD, 400 MHz)

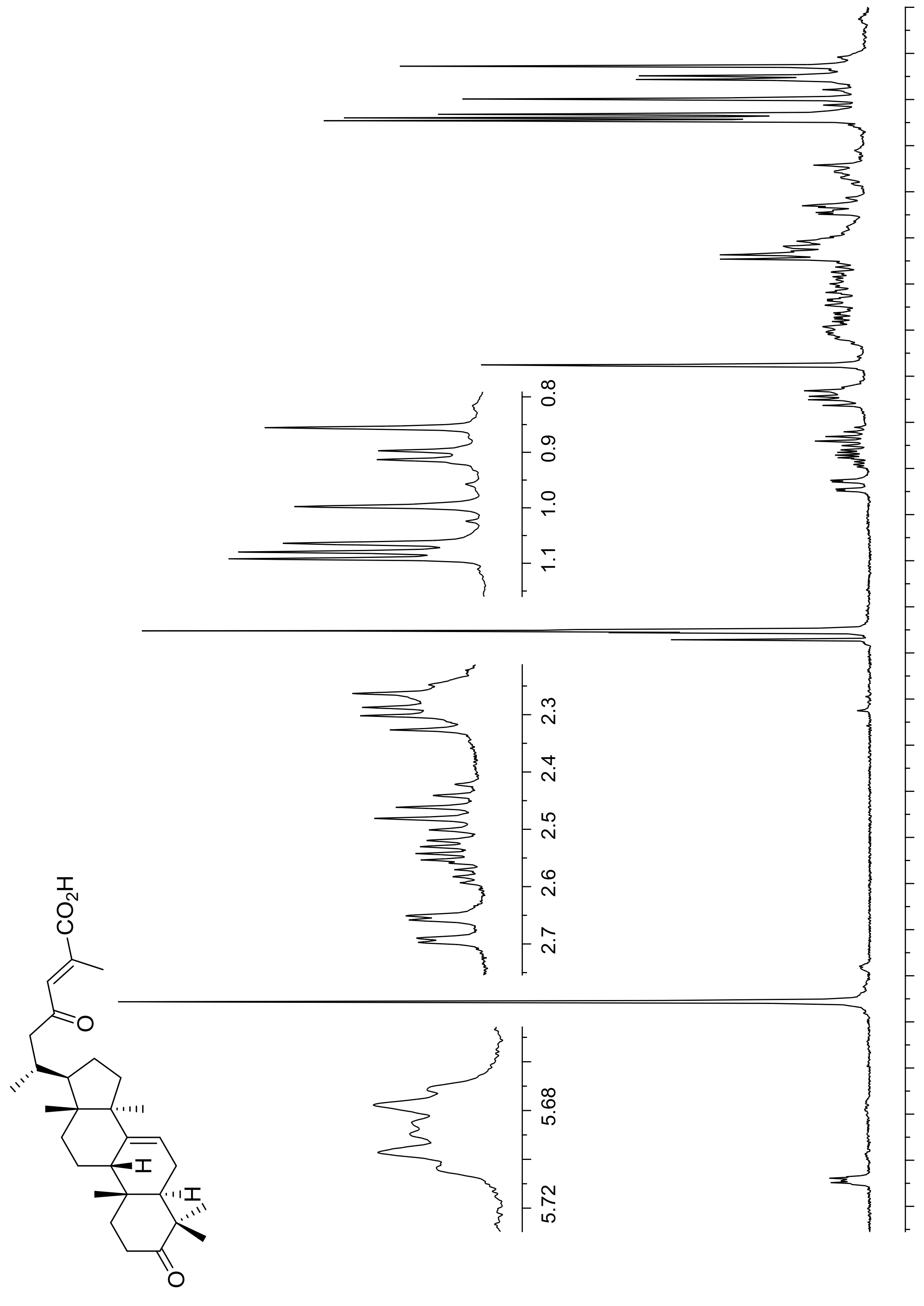

^{13}C NMR spectrum of **7** (CD_3OD, 100 MHz)

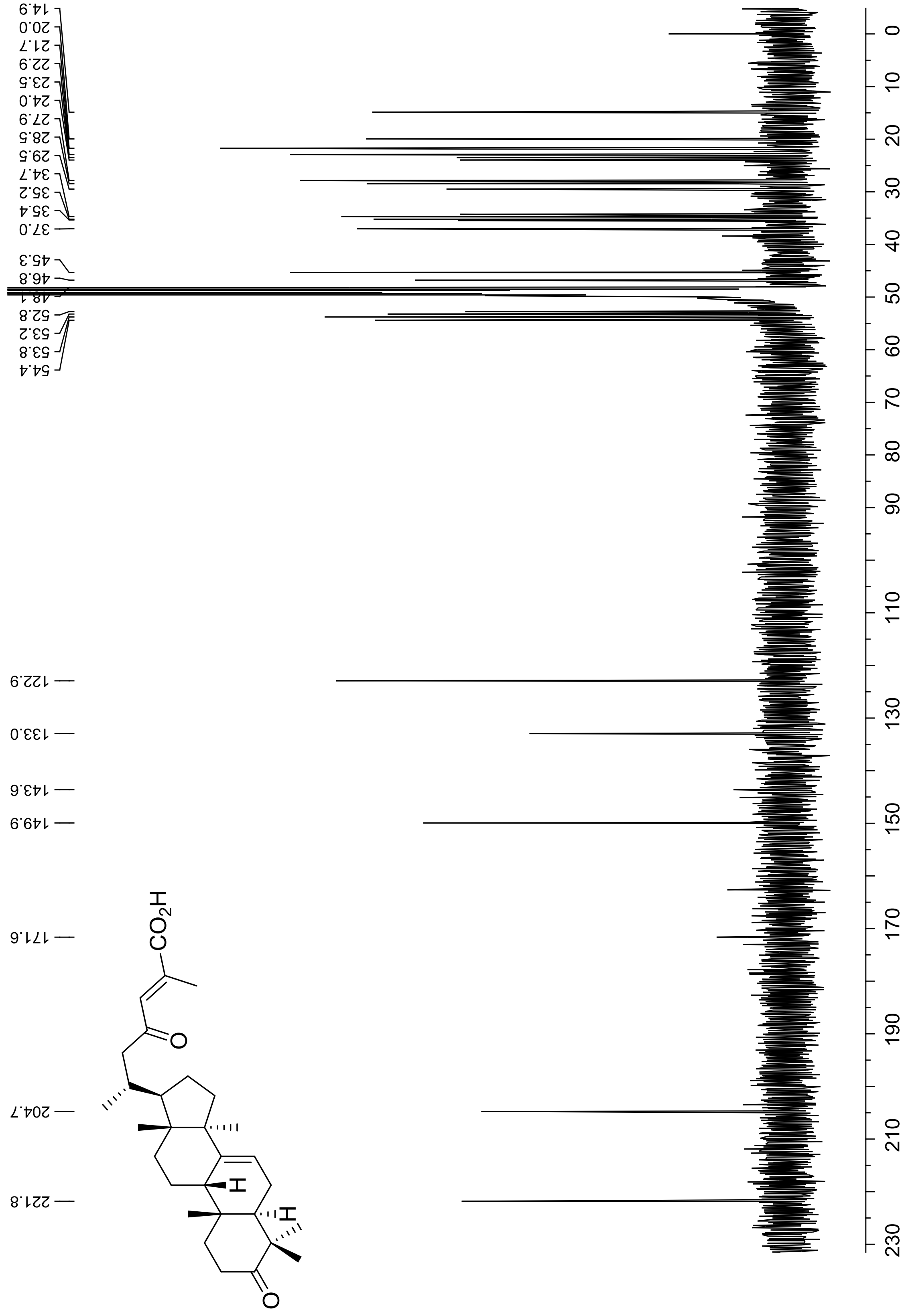

^{1}H NMR spectrum of **8** ($CDCl_3$, 400 MHz)

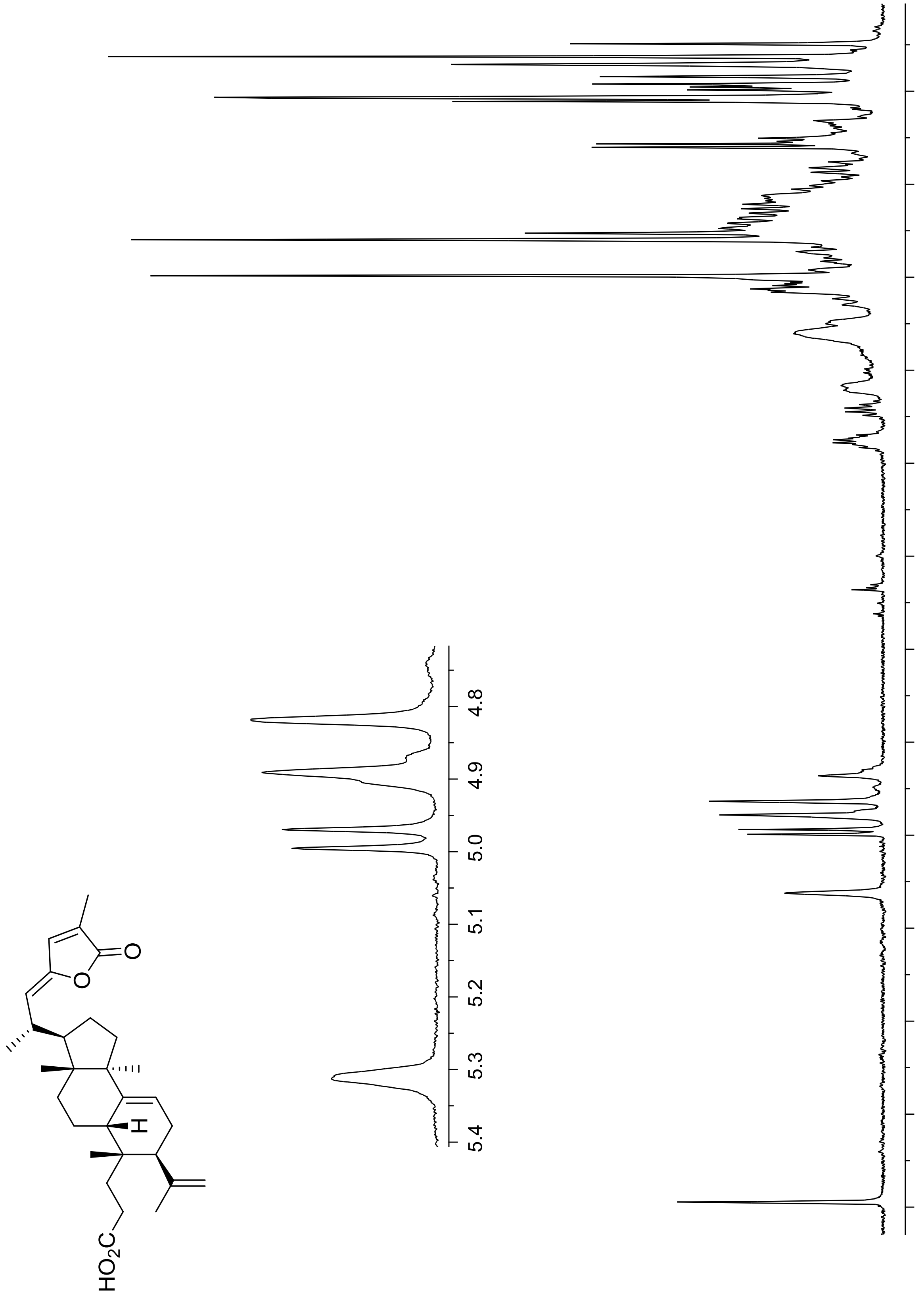

^{13}C NMR spectrum of **8** ($CDCl_3$, 100 MHz)

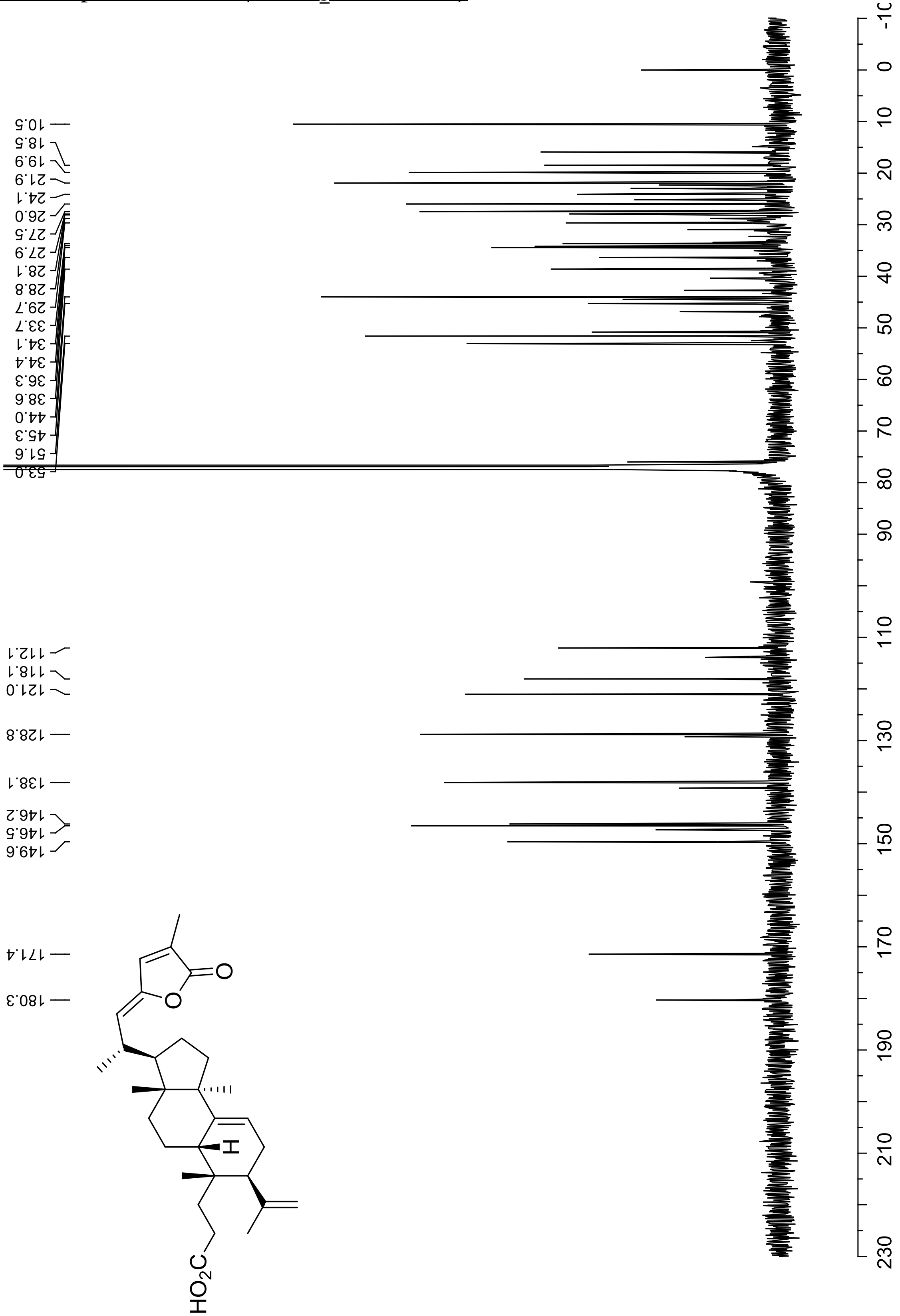

^{1}H NMR spectrum of **9** ($CDCl_3$, 400 MHz)

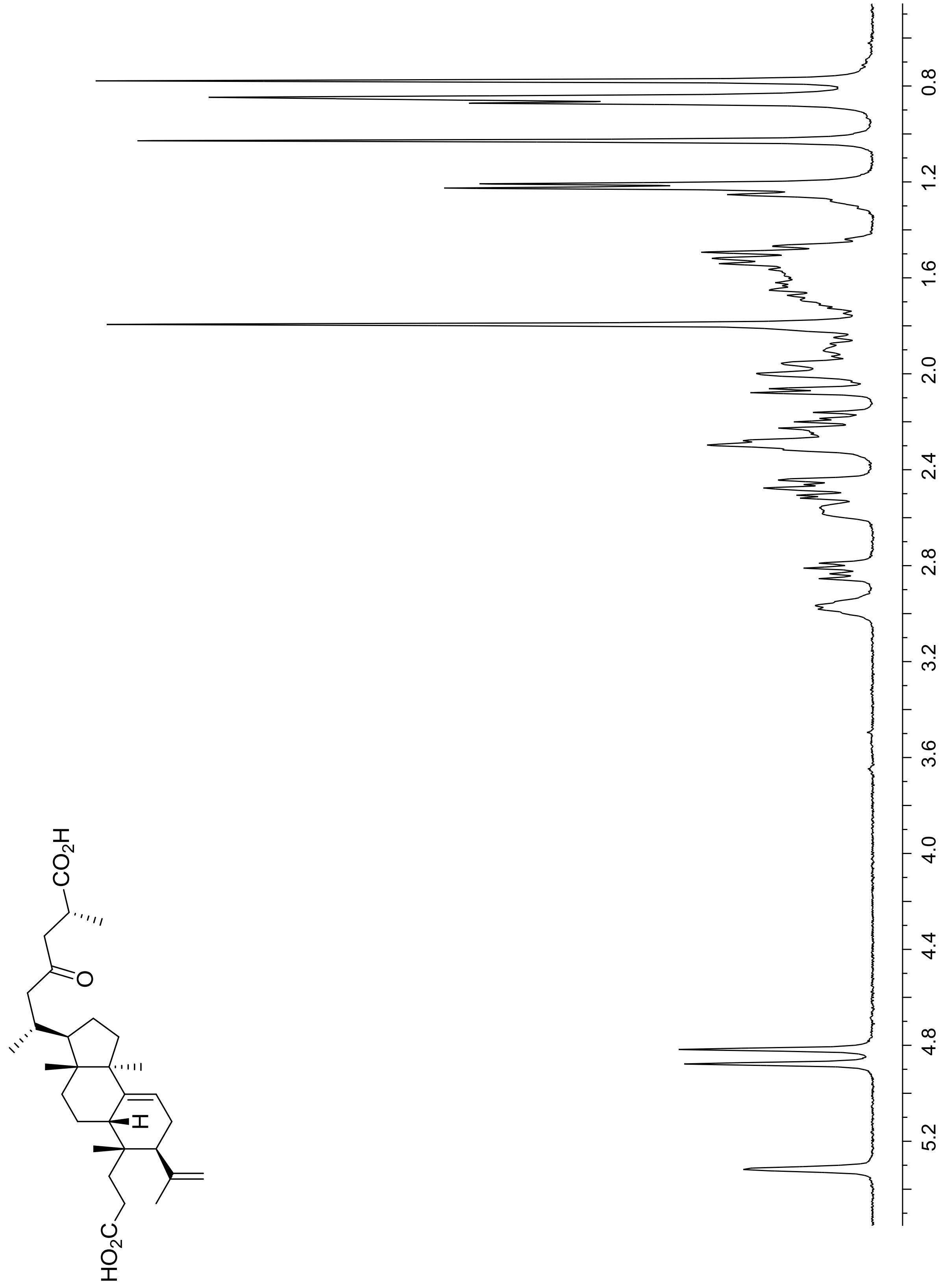

^{13}C NMR spectrum of **9** ($CDCl_3$, 100 MHz)

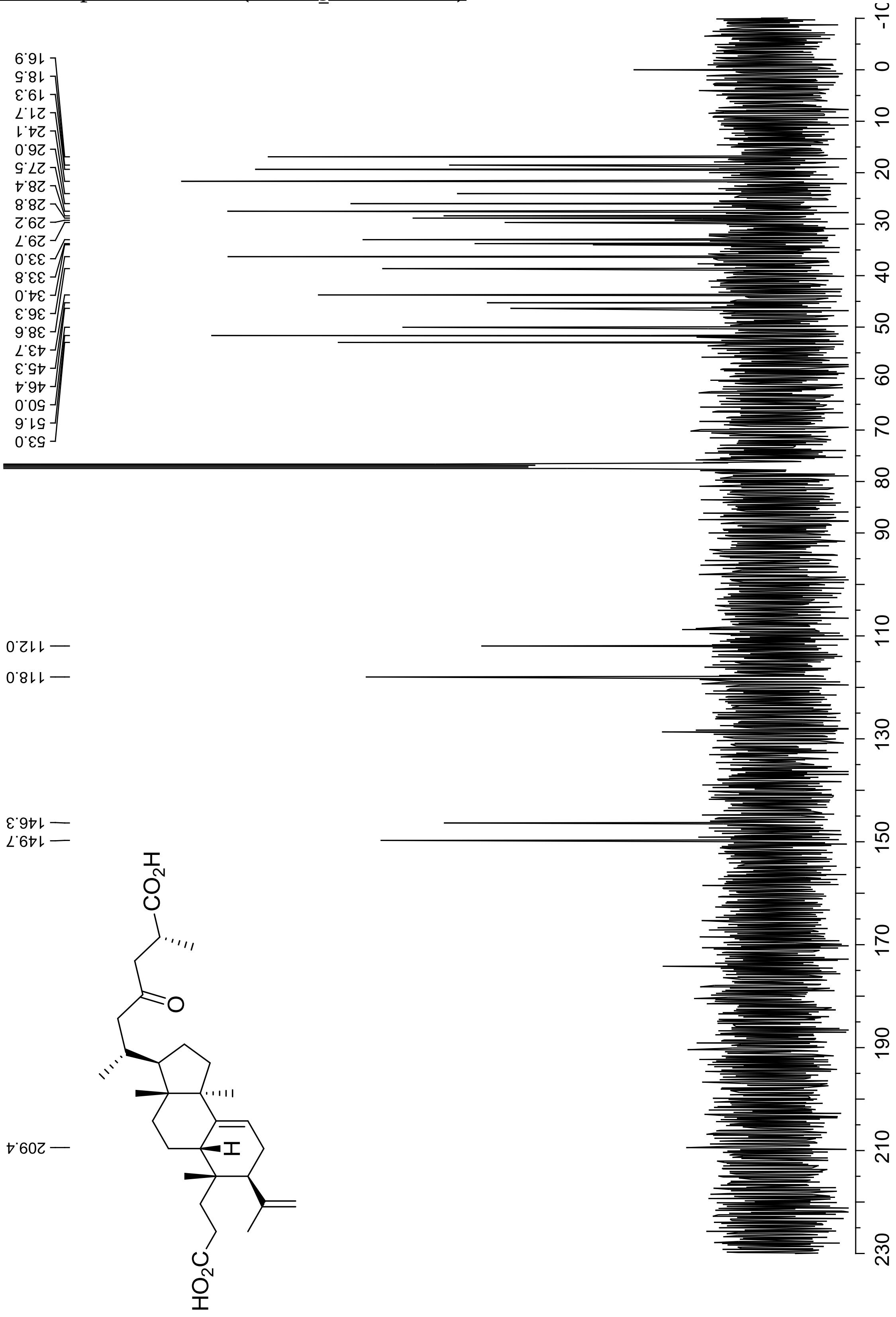

^{1}H NMR spectrum of **10** ($CDCl_3$, 400 MHz)

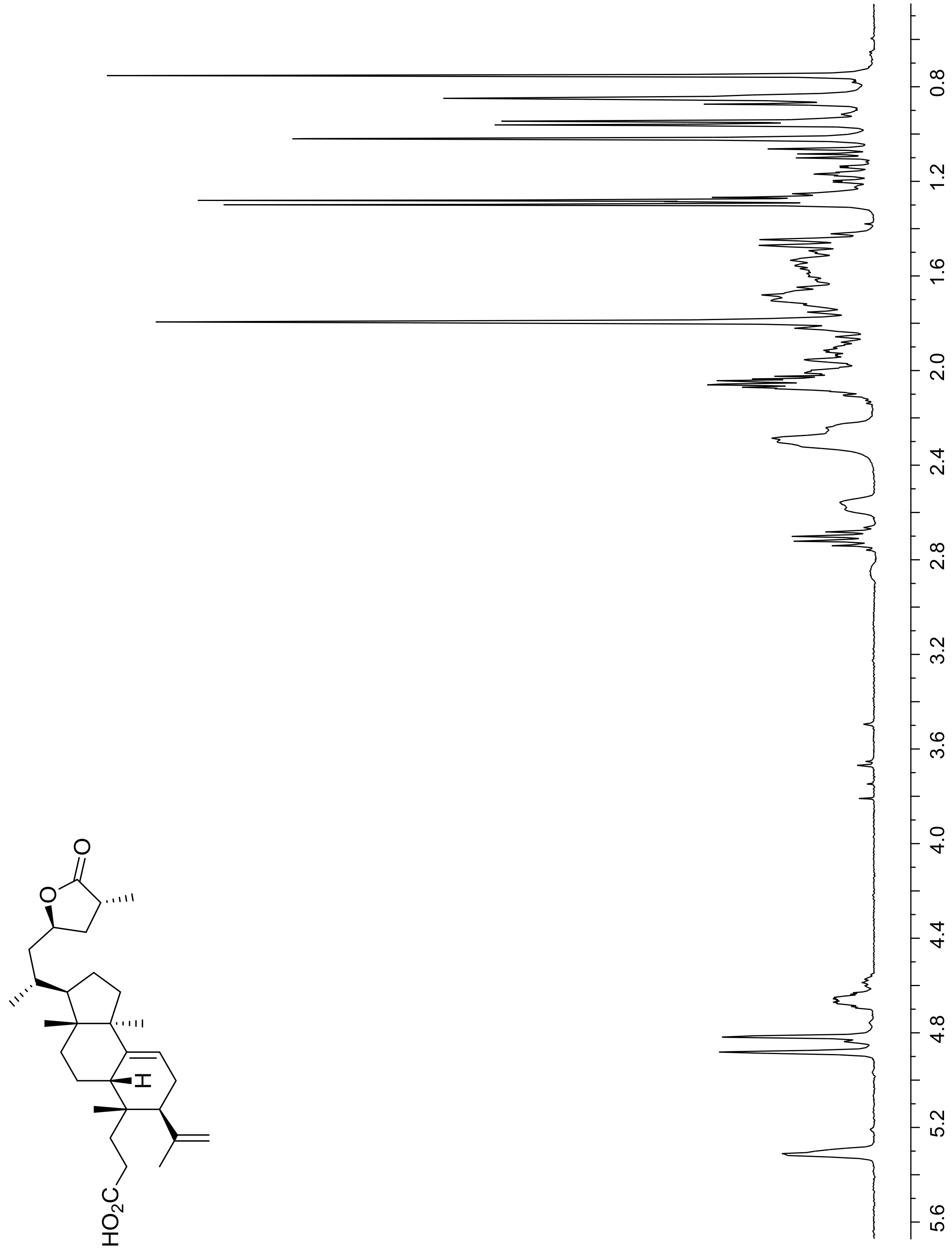

^{13}C NMR spectrum of **10** ($CDCl_3$, 100 MHz)

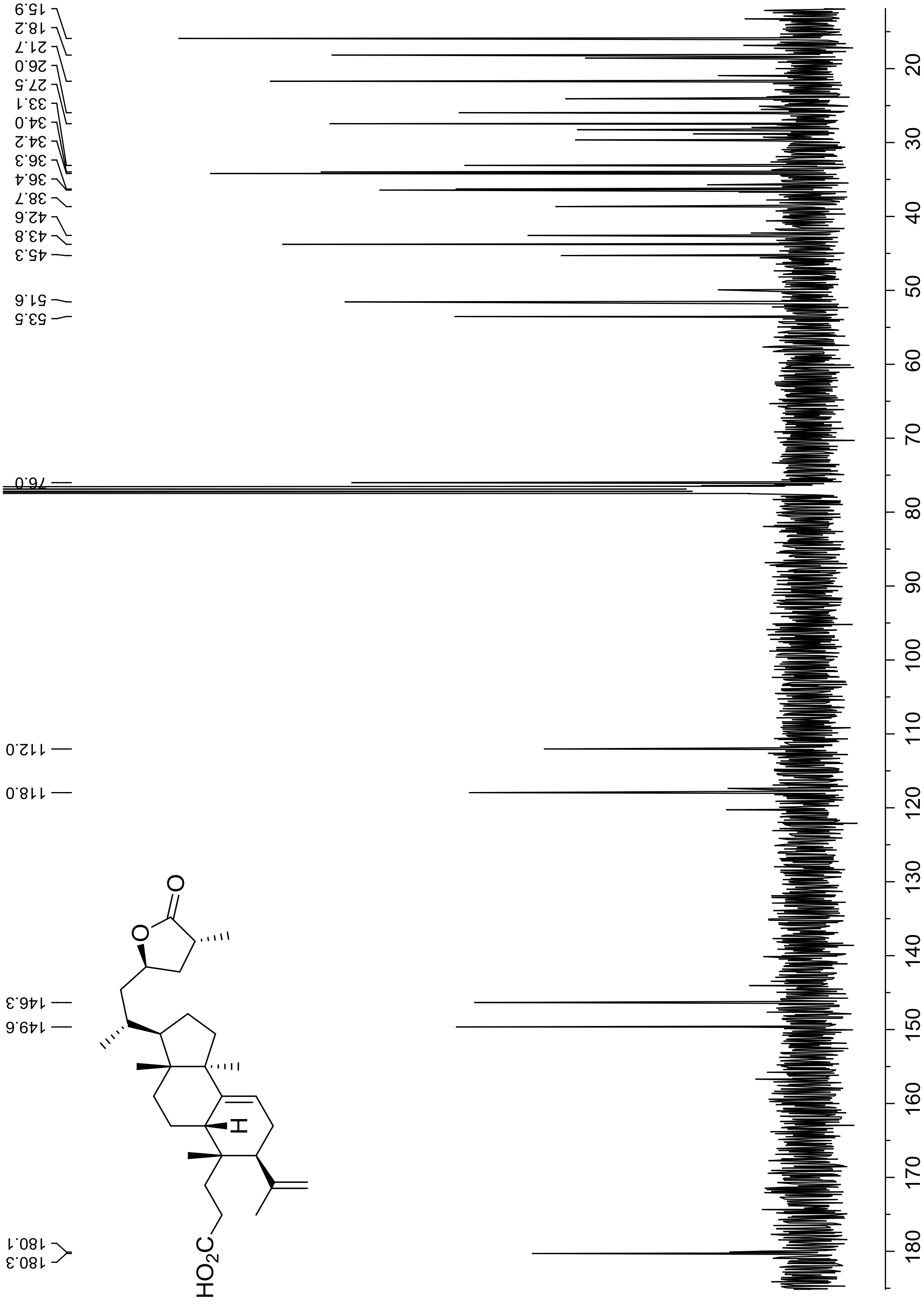

^{1}H NMR spectrum of **11** ($CDCl_3$, 400 MHz)

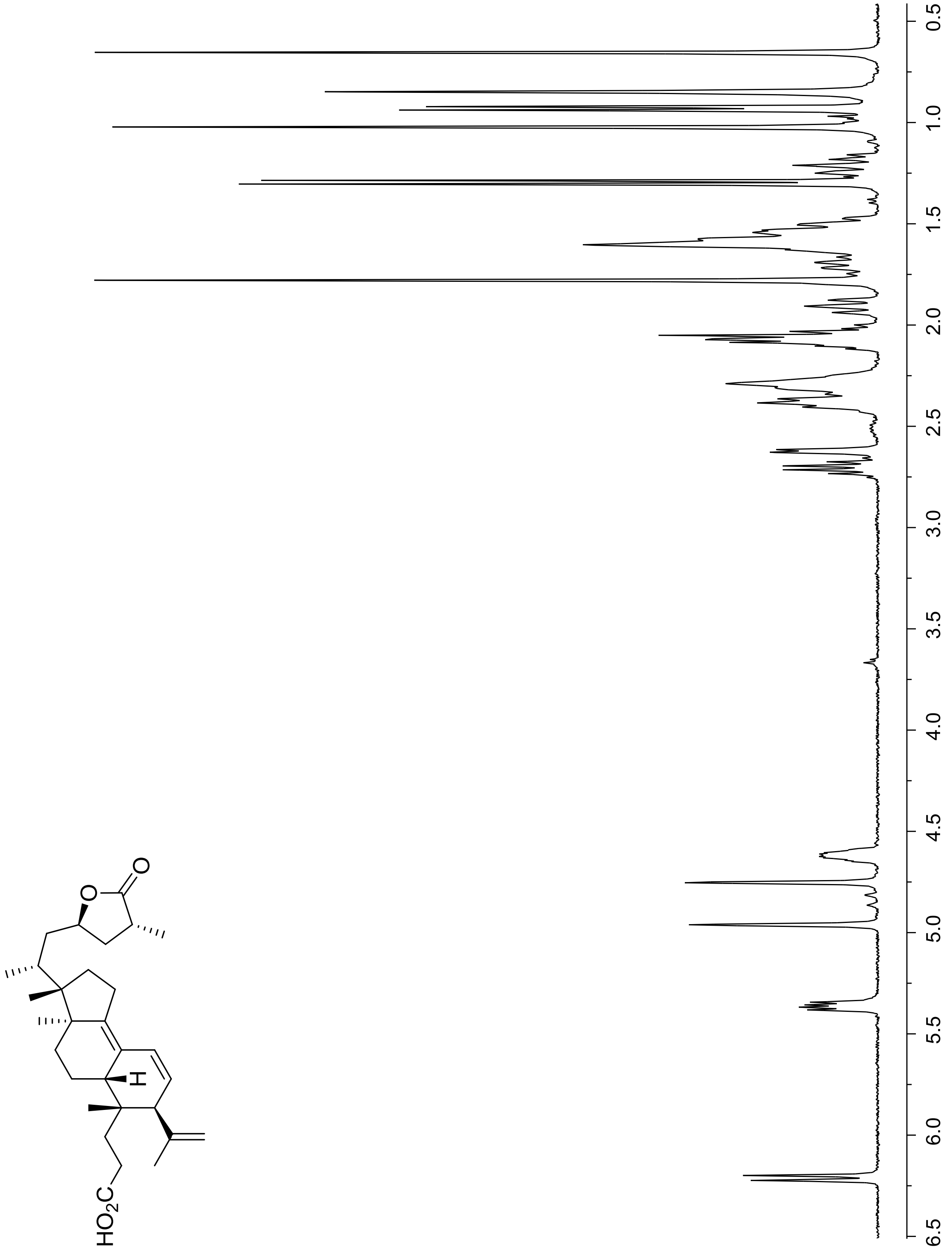

^{13}C NMR spectrum of **11** ($CDCl_3$, 100 MHz)

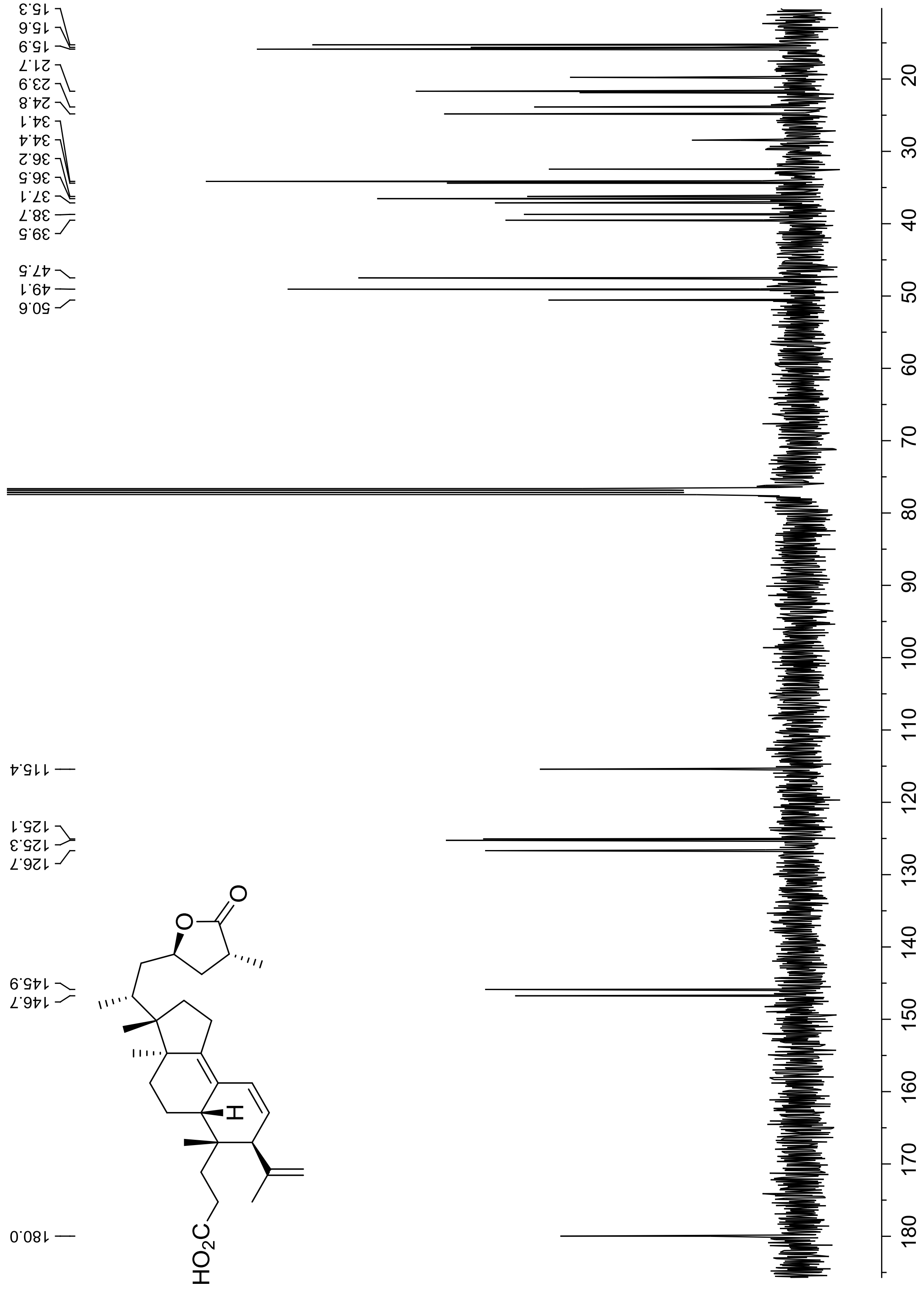

^{1}H NMR spectrum of **12** ($CDCl_3$/CD_3OD 1:1, 400 MHz)

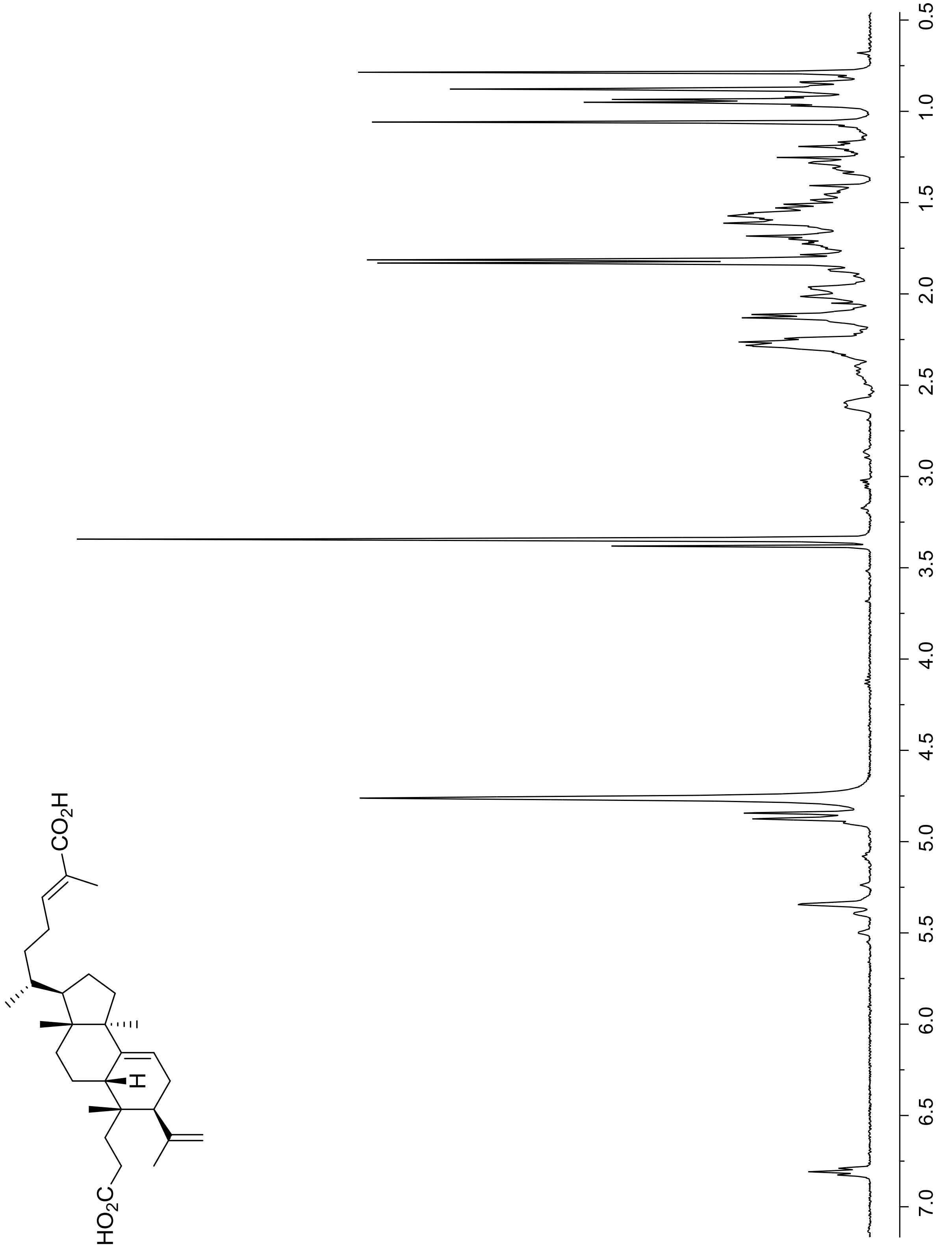

^{13}C NMR spectrum of **12** ($CDCl_3/CD_3OD$ 1:1, 100 MHz)

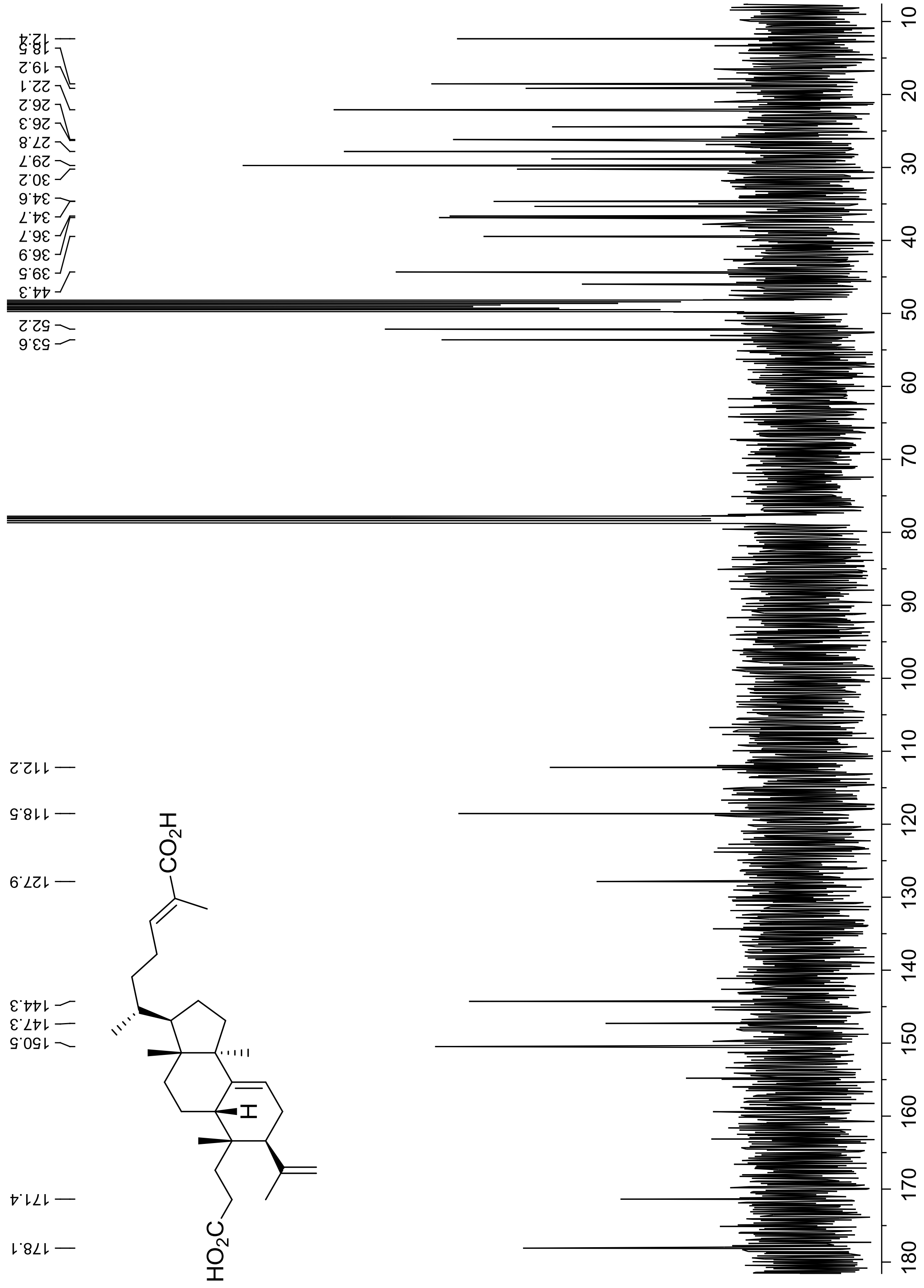

^{1}H NMR spectrum of **13** ($CDCl_3$, 400 MHz)

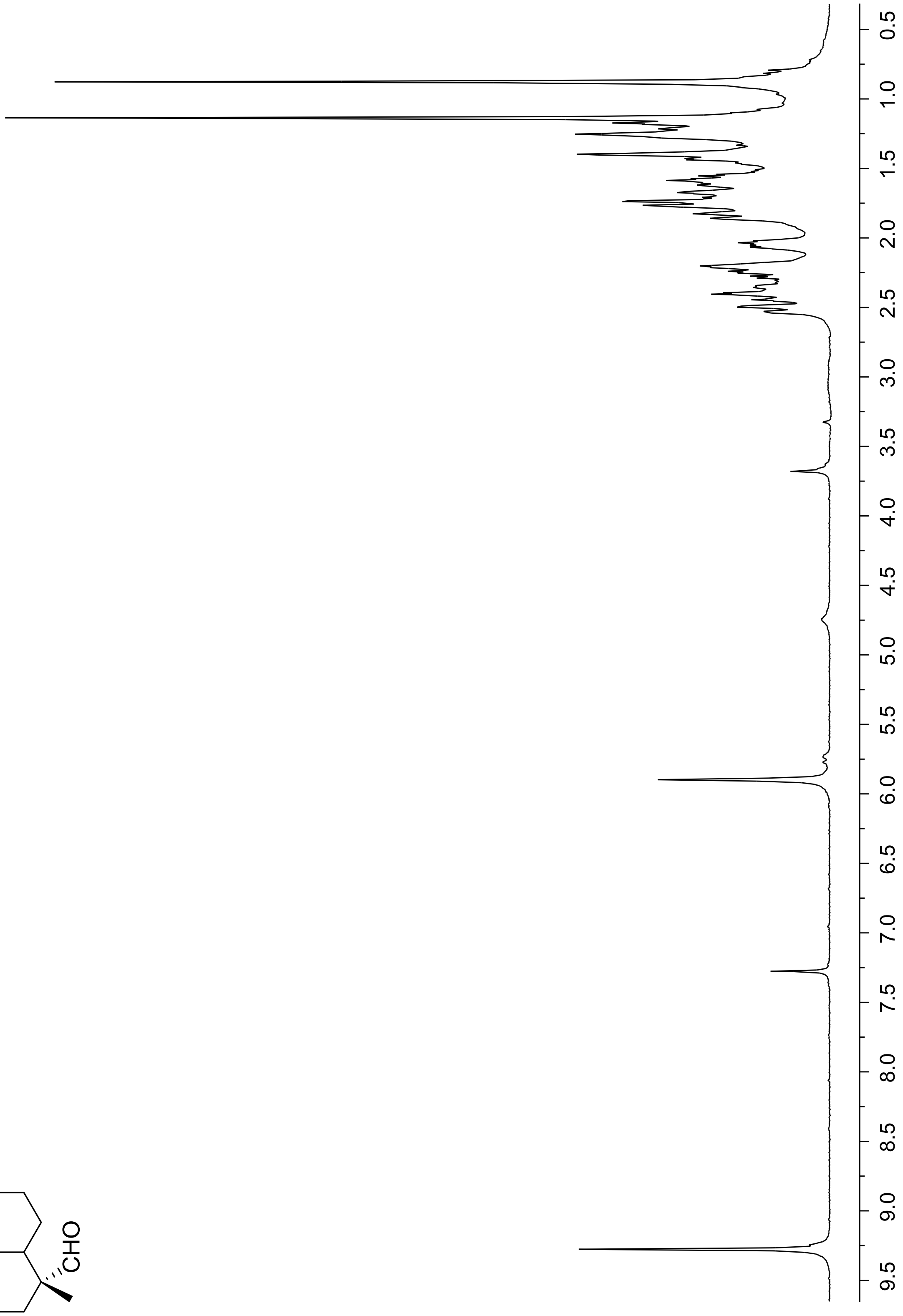

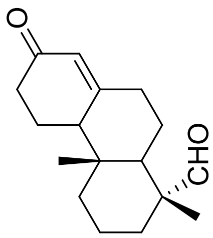

^{13}C NMR spectrum of **13** ($CDCl_3$, 100 MHz)

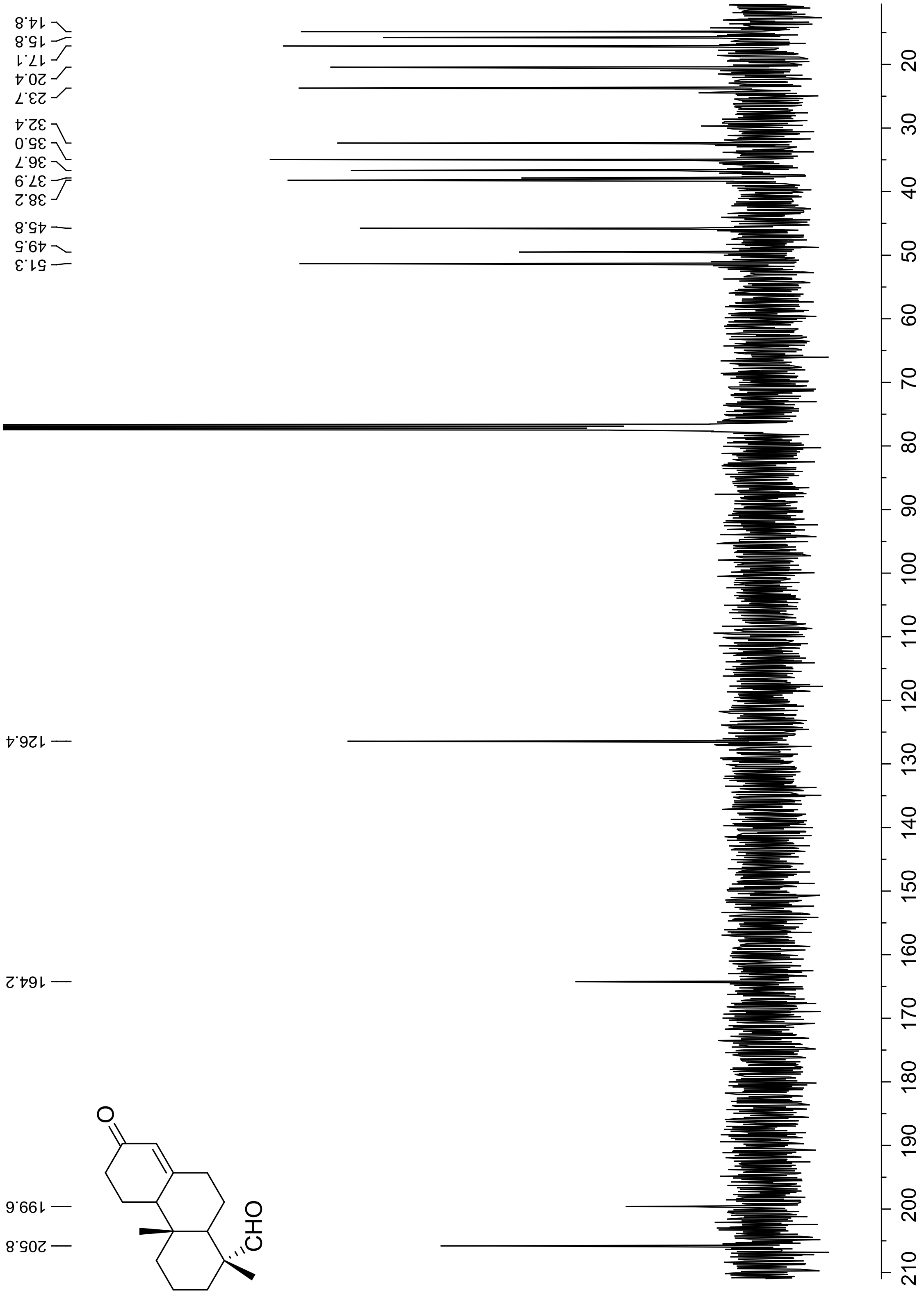

^{1}H NMR spectrum of **14** ($CDCl_3$, 400 MHz)

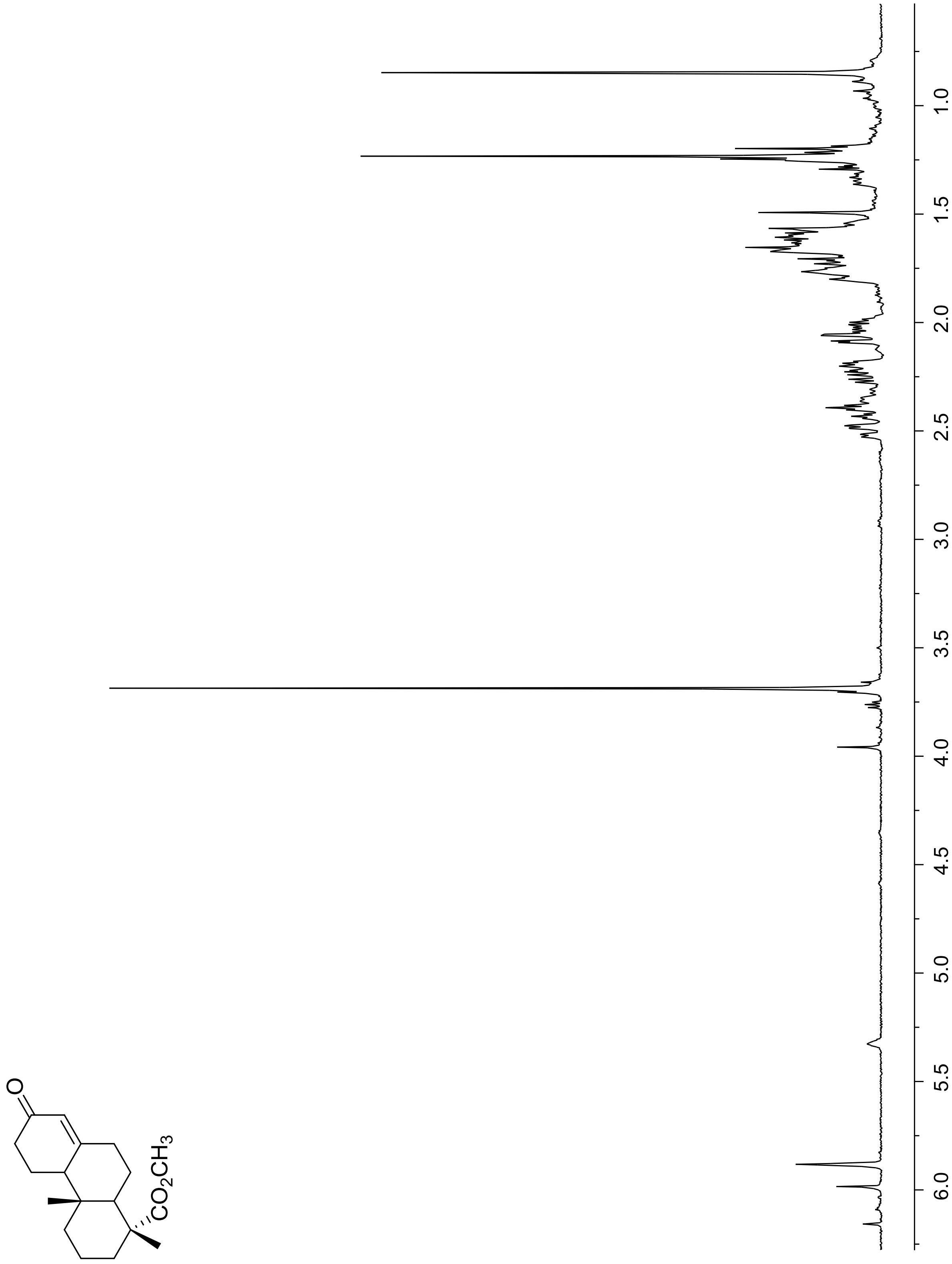

^{13}C NMR spectrum of **14** ($CDCl_3$, 100 MHz)

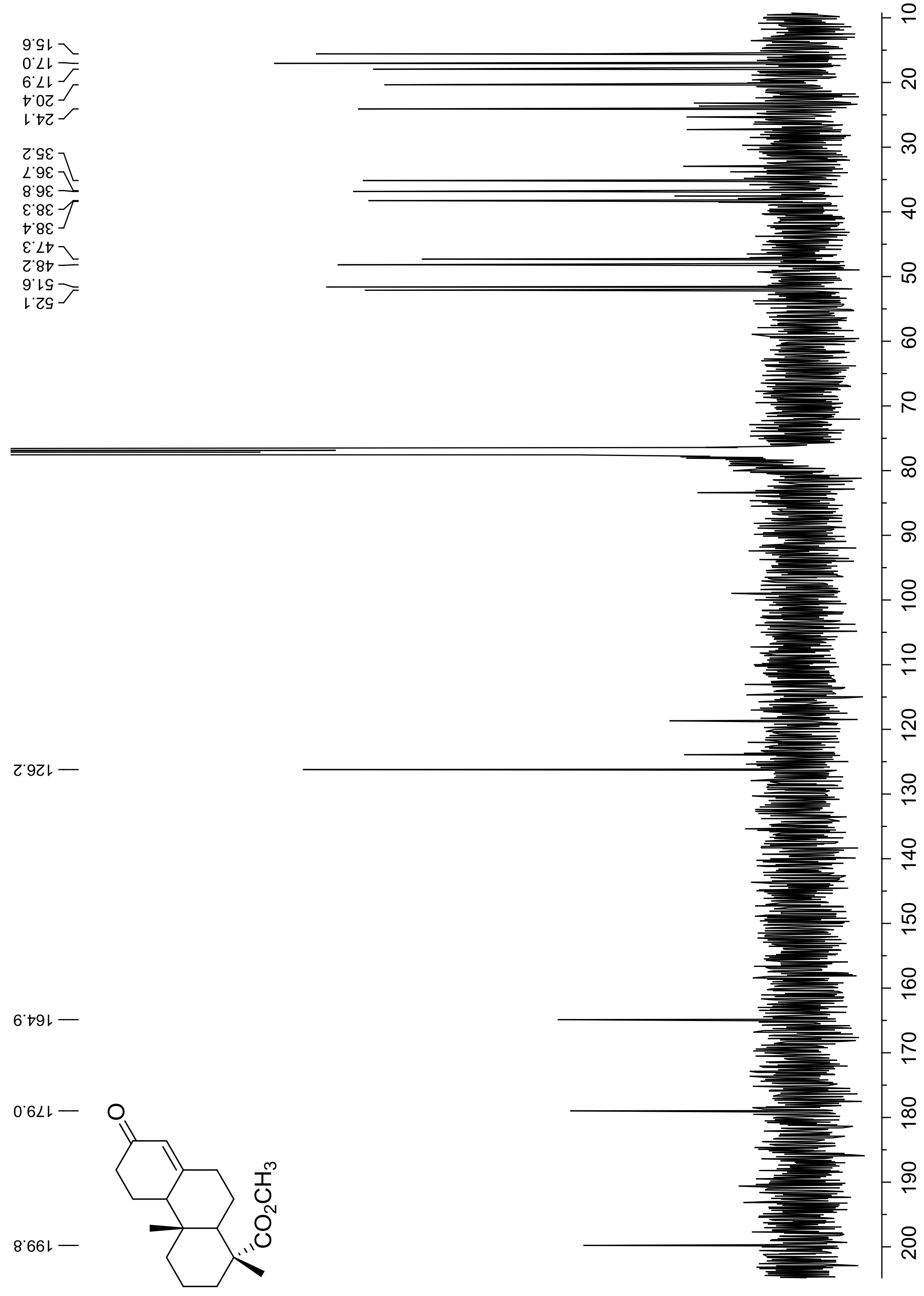

^{1}H NMR spectrum of **15** ($CDCl_3$, 400 MHz)

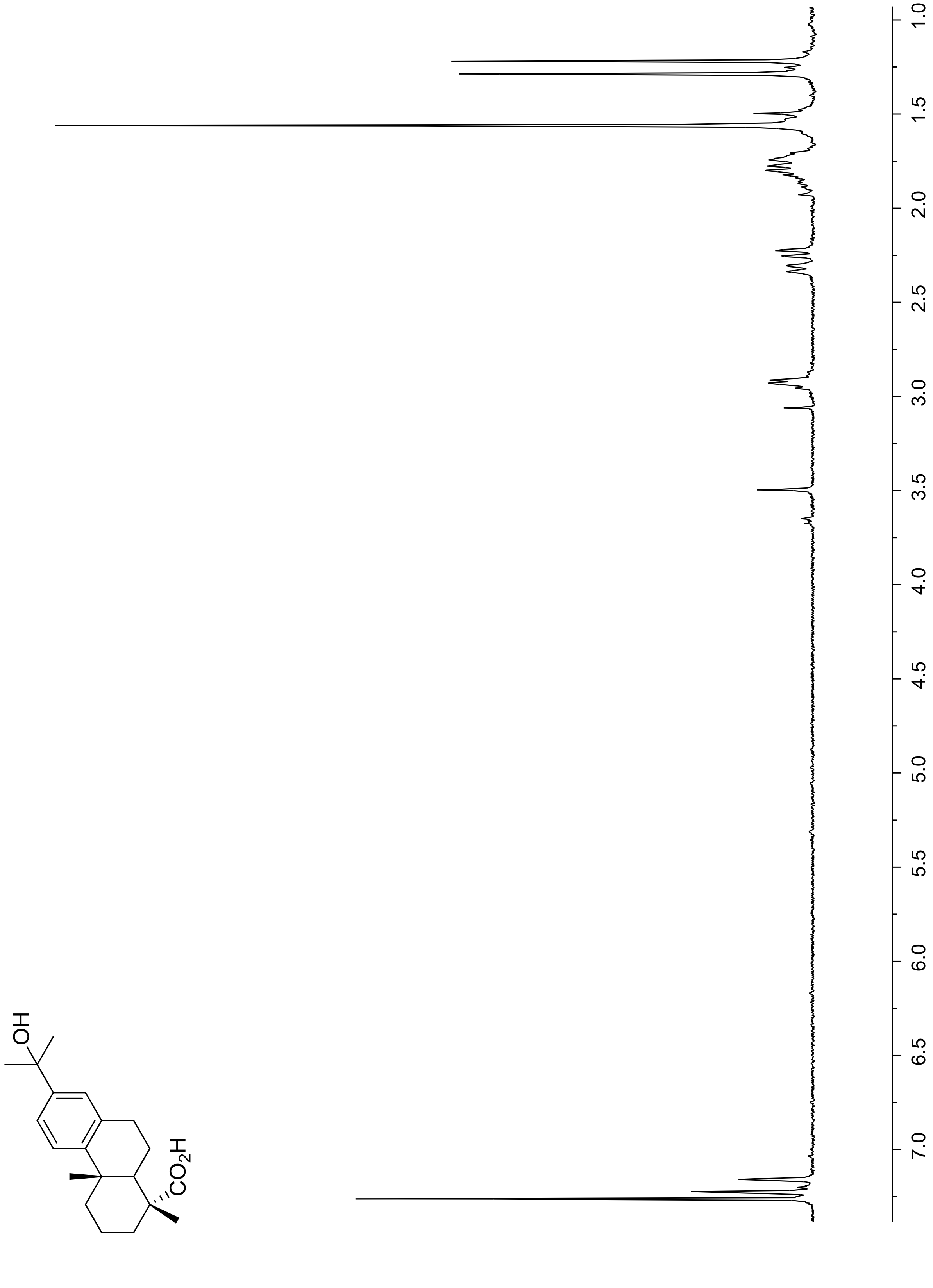

^{13}C NMR spectrum of **15** ($CDCl_3$, 100 MHz)

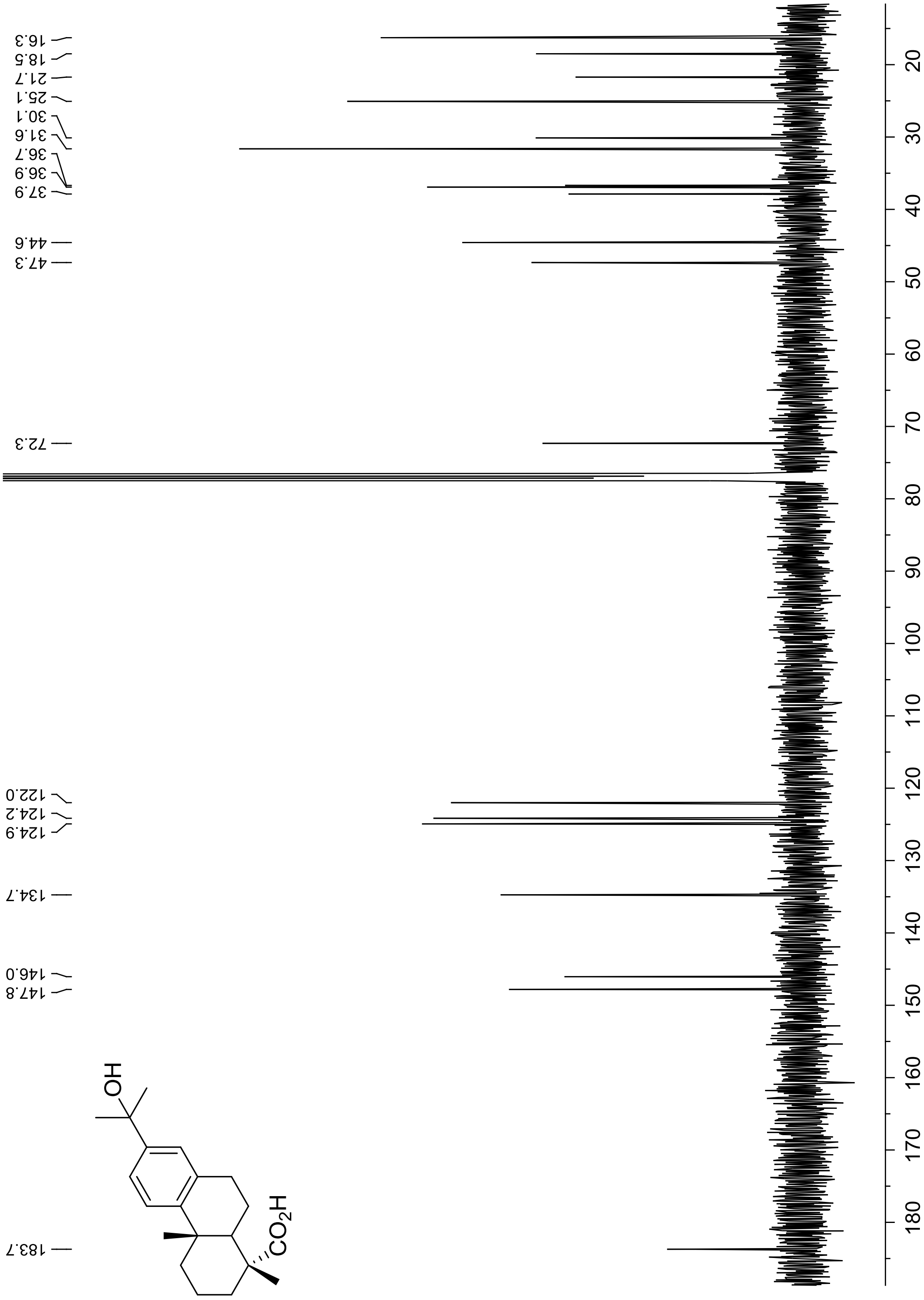

^{1}H NMR spectrum of **16** ($CDCl_3$, 400 MHz)

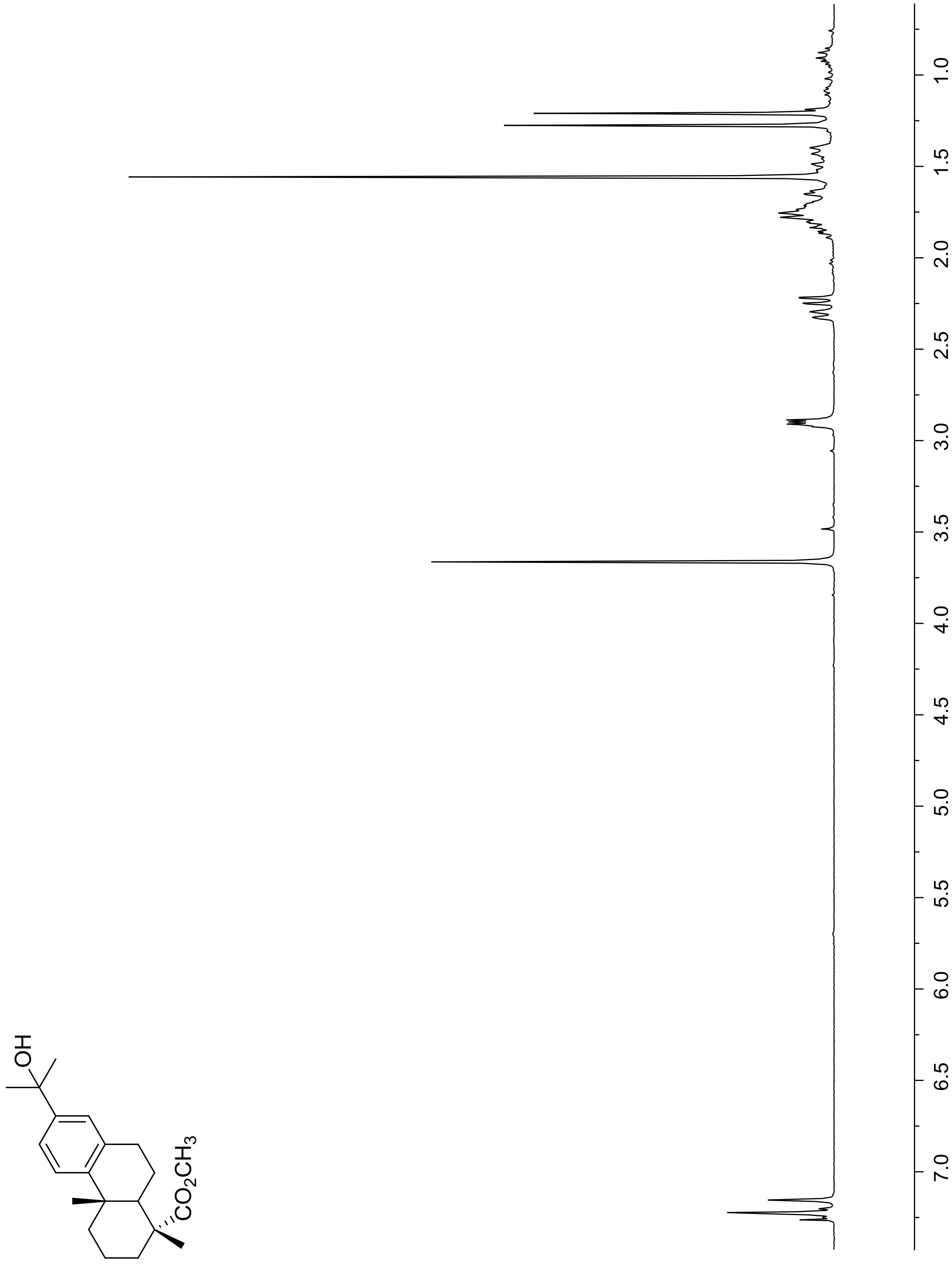

^{13}C NMR spectrum of **16** ($CDCl_3$, 100 MHz)

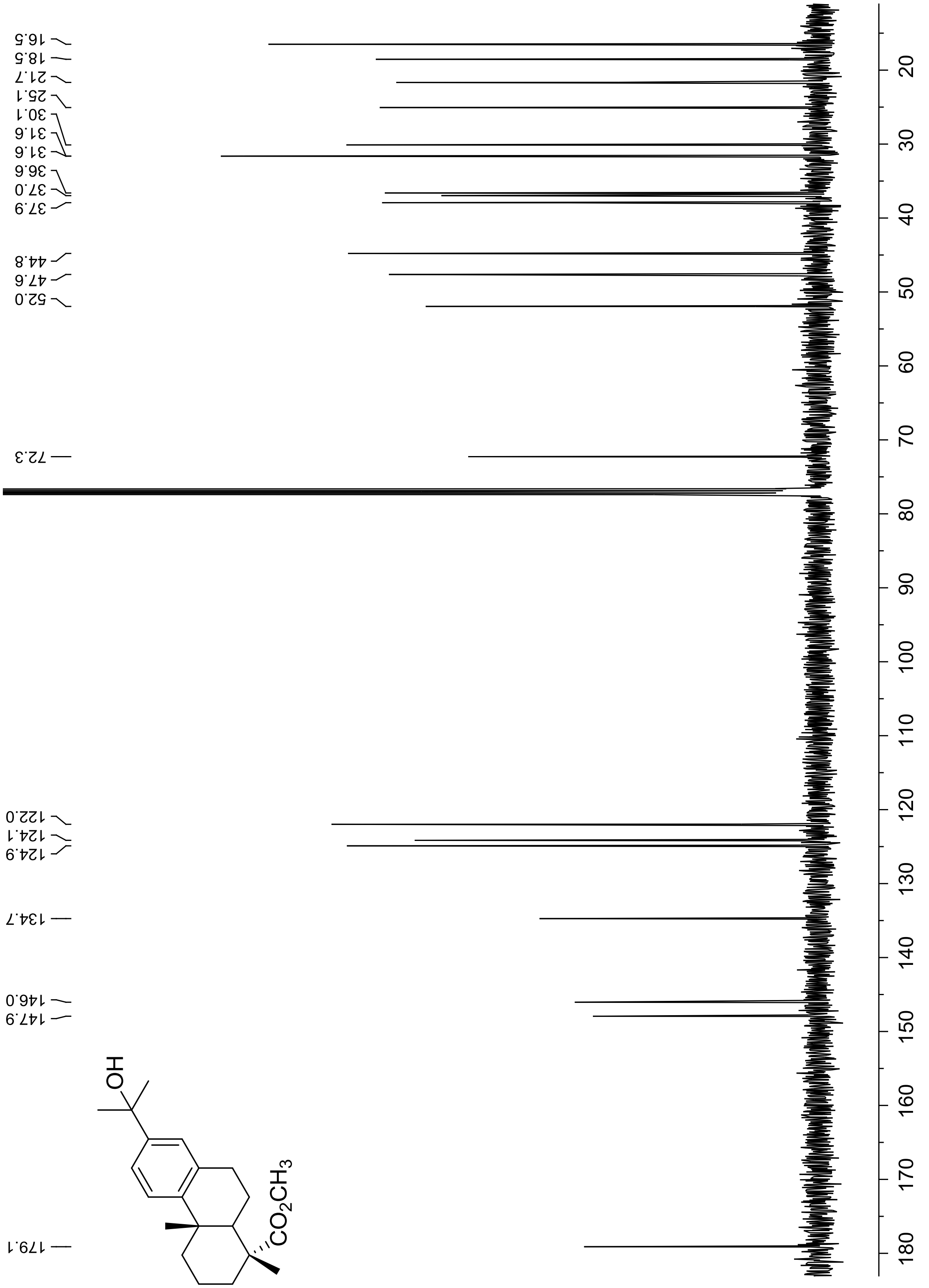

^{1}H NMR spectrum of **17** (CD_3OD, 400 MHz)

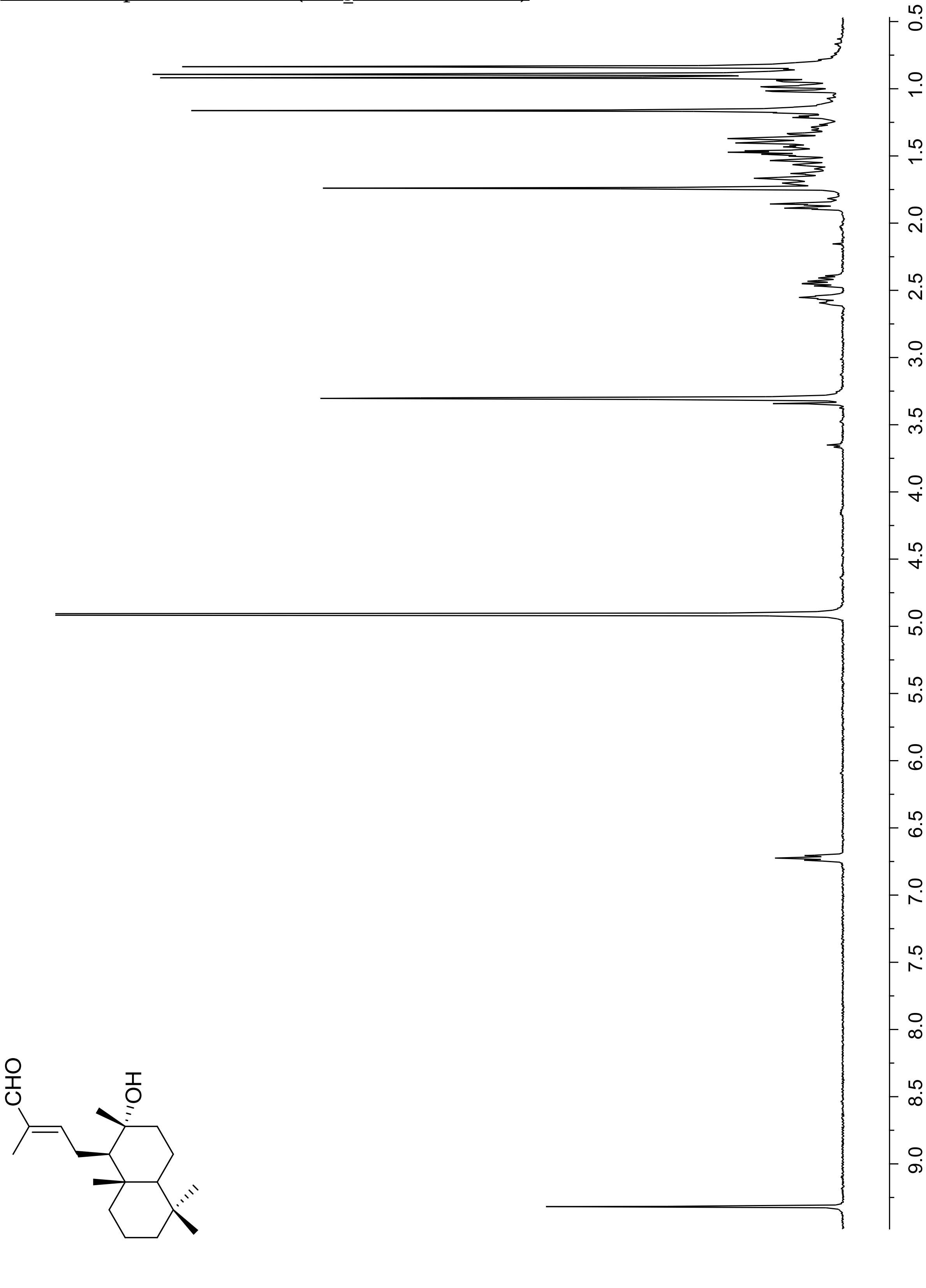

^{13}C NMR spectrum of **17** (CD_3OD, 100 MHz)

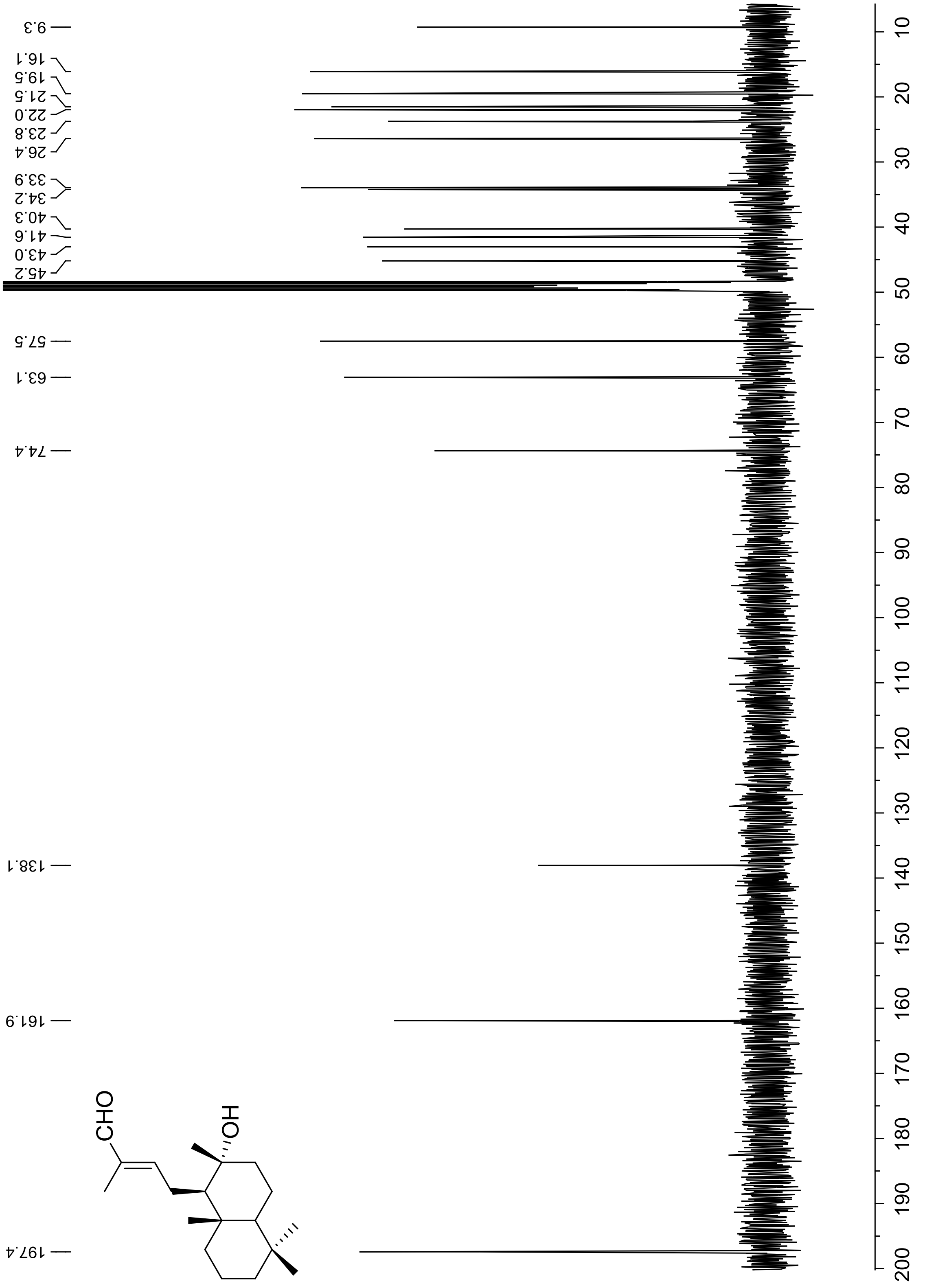

^{1}H NMR spectrum of **18** ($CDCl_3$, 400 MHz)

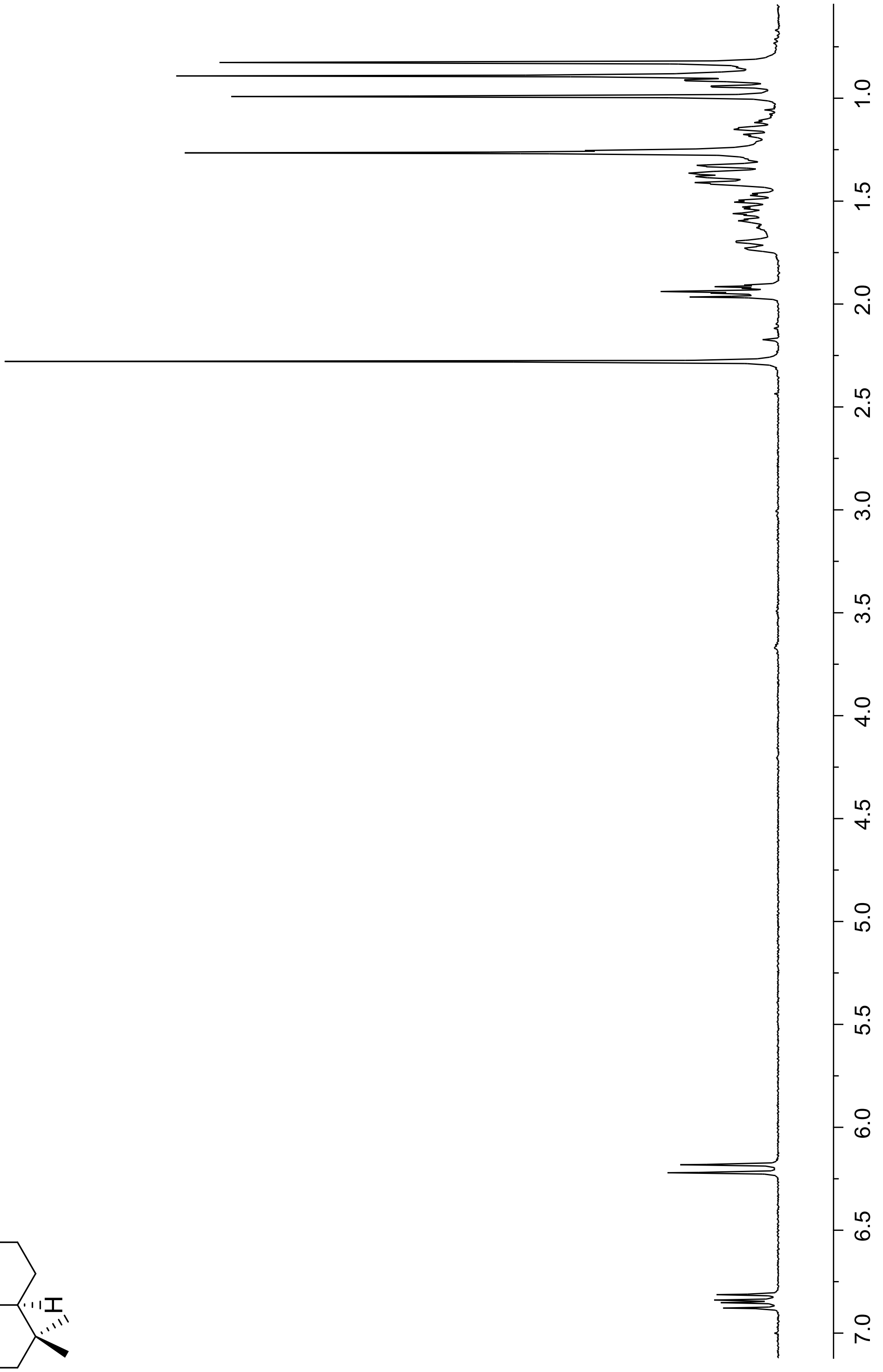

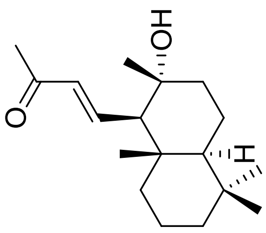

^{13}C NMR spectrum of **18** ($CDCl_3$, 100 MHz)

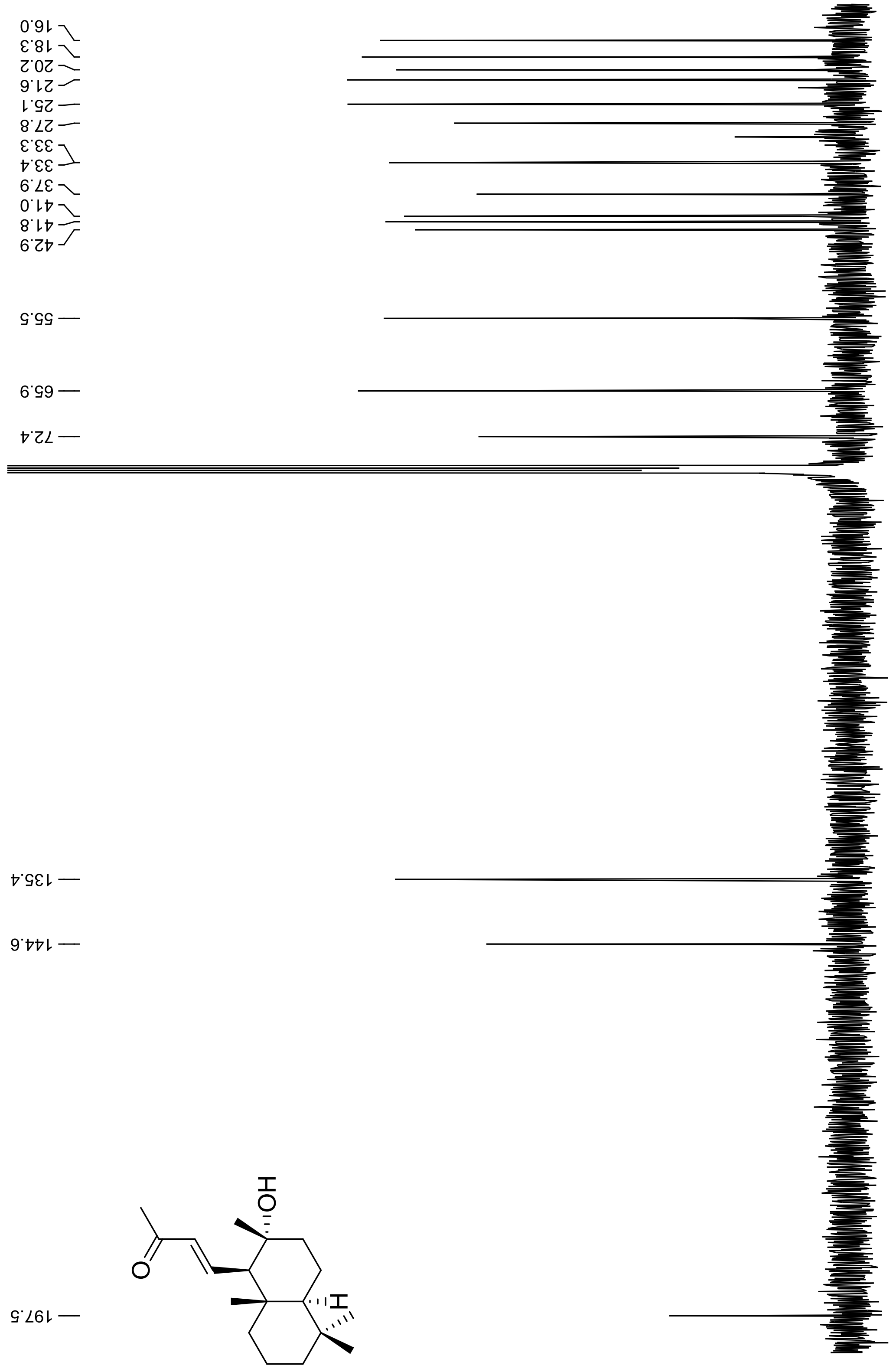

Supplement: File 1 — Experimental procedures, product characterization and 1H and 13C spectra for compounds 1–18. [file Beilstein_J_Org_Chem-09-1333-s001.pdf]
